# Integrative analysis of RUNX1 downstream pathways and target genes

**DOI:** 10.1186/1471-2164-9-363

**Published:** 2008-07-31

**Authors:** Joëlle Michaud, Ken M Simpson, Robert Escher, Karine Buchet-Poyau, Tim Beissbarth, Catherine Carmichael, Matthew E Ritchie, Frédéric Schütz, Ping Cannon, Marjorie Liu, Xiaofeng Shen, Yoshiaki Ito, Wendy H Raskind, Marshall S Horwitz, Motomi Osato, David R Turner, Terence P Speed, Maria Kavallaris, Gordon K Smyth, Hamish S Scott

**Affiliations:** 1 Molecular Medicine Division, The Walter and Eliza Hall Institute of Medical Research, Parkville 3050, Victoria, Australia; 2 Department of Medical Biology, The University of Melbourne, 3050 Parkville, Victoria, Australia; 3 Bioinformatics Division, The Walter and Eliza Hall Institute of Medical Research, Parkville 3050, Victoria, Australia; 4 Division of Medical Genetics, University of Geneva Medical School, 1211 Geneva, Switzerland; 5 Experimental Therapeutics Program, Children's Cancer Institute Australia for Medical Research, 2031 NSW, Australia; 6 Department of Hematology and Genetic Pathology, School of Medicine, Flinders University, 5001 South Australia, Australia; 7 Molecular and Cell Biology, National University of Singapore, 117543 Singapore; 8 Division of Medical Genetics, University of Washington, Seattle, USA; 9 Center for Integrative Genomics, University of Lausanne, Switzerland; 10 Internal Medicine, University Hospital, Berne, Switzerland; 11 Université de Lyon, Lyon, F-69008, France; Université Lyon 1, Domaine Rockfeller, Lyon, F-69008, France; 12 CNRS UMR 5201, Laboratoire de Génétique Moléculaire, Signalisation et Cancer, Lyon, F-69008, France; 13 Division of Molecular Genome Analysis, German Cancer Research Center (DKFZ), Heidelberg, Germany; 14 Division of Molecular Pathology, the Institute of Medical and Veterinary Science and The Hanson Institute, Box 14 Rundle Mall Post Office, Adelaide, SA 5000, Australia; 15 The School of Medicine the University of Adelaide, SA, 5005, Australia

## Abstract

**Background:**

The *RUNX1 *transcription factor gene is frequently mutated in sporadic myeloid and lymphoid leukemia through translocation, point mutation or amplification. It is also responsible for a familial platelet disorder with predisposition to acute myeloid leukemia (FPD-AML). The disruption of the largely unknown biological pathways controlled by RUNX1 is likely to be responsible for the development of leukemia. We have used multiple microarray platforms and bioinformatic techniques to help identify these biological pathways to aid in the understanding of why RUNX1 mutations lead to leukemia.

**Results:**

Here we report genes regulated either directly or indirectly by RUNX1 based on the study of gene expression profiles generated from 3 different human and mouse platforms. The platforms used were global gene expression profiling of: 1) cell lines with RUNX1 mutations from FPD-AML patients, 2) over-expression of RUNX1 and CBFβ, and 3) Runx1 knockout mouse embryos using either cDNA or Affymetrix microarrays. We observe that our datasets (lists of differentially expressed genes) significantly correlate with published microarray data from sporadic AML patients with mutations in either *RUNX1 *or its cofactor, *CBFβ*. A number of biological processes were identified among the differentially expressed genes and functional assays suggest that heterozygous *RUNX1 *point mutations in patients with FPD-AML impair cell proliferation, microtubule dynamics and possibly genetic stability. In addition, analysis of the regulatory regions of the differentially expressed genes has for the first time systematically identified numerous potential novel RUNX1 target genes.

**Conclusion:**

This work is the first large-scale study attempting to identify the genetic networks regulated by RUNX1, a master regulator in the development of the hematopoietic system and leukemia. The biological pathways and target genes controlled by RUNX1 will have considerable importance in disease progression in both familial and sporadic leukemia as well as therapeutic implications.

## Background

The Core Binding Factor (CBF) is a transcriptional regulator complex, which is composed of two sub-units [1]. Mammals have three genes coding for the α-subunits, *RUNX1*, *RUNX2 *and *RUNX3 *[2], and one coding for the β-subunit, *CBFβ *. The α-subunits recognize a specific sequence (TGT/cGGT) in the regulatory regions of their target genes in order to bind DNA directly, while the β-subunit heterodimerizes with the α-subunits but does not interact directly with the DNA. The interaction with CBFβ stabilizes the RUNX-DNA complex [3,4] and protects the RUNX proteins from degradation [5].

In humans, the CBF complex containing RUNX1 as the α-subunit is one of the most frequent targets of chromosomal and genetic alterations in leukemia. Chromosomal rearrangements involving *RUNX1 *or *CBFβ *[6], somatic point mutations in *RUNX1 *[7] and amplification of *RUNX1 *[8] have all been described in acute leukemia. In addition to somatic alterations, germ-line point mutations in *RUNX1 *are responsible for an autosomal dominant platelet disorder with a propensity to develop leukemia (FPD-AML, OMIM 601399) [9,10]. Interestingly, the dosage of RUNX1 protein seems to play a role in the determination of the leukemic phenotype. Indeed, low dosage of RUNX1, resulting from haploinsufficient or dominant negative mutations, lead to the development of myeloid leukemia [9-11], whereas amplification of *RUNX1 *gene is more often observed in lymphoid leukemia, particularly pediatric ALL [12]. A number of observations also suggest that although *RUNX1 *is involved in the first steps of leukemia development, additional somatic mutations are necessary and probably determinant for the leukemic phenotype: 1) The predisposition to develop leukemia in FPD-AML patients shows that germline *RUNX1 *mutations are not sufficient for the development of the disease [10]. 2) Somatic translocations are not able to induce leukemia in mouse cells on their own [13]. 3) The translocation t(12;21), which fuses *ETV6 (TEL) *to *RUNX1*, can arise *in utero *but does not trigger leukemia until later in childhood, with as much as nine years latency [14]. These additional mutations are likely to occur in molecules involved in the same biological pathways as RUNX1, as hemizygous loss of several molecules in the same biological pathway (e.g. RUNX1 and SPI1) is thought to be almost as tumorigenic as homozygous loss of one molecule (e.g. homozygous RUNX1 mutation in AML-M0) [15]. Therefore the identification of downstream targets of RUNX1, with care to the model systems including species and cell type of origin, is of great interest in order to identify novel candidate molecules involved in leukemogenesis.

The identification of the biological pathways regulated by RUNX1 is also of importance to shed light on its *in vivo *function and role in leukemia development. The observation that *Runx1 *knockout mice show a lack of definitive hematopoietic maturation and die at embryonic stage 12 from hemorrhages in the central nervous system demonstrates that RUNX1 plays a critical role during development of the hematopoietic system [16,17]. In addition, RUNX1 might also play a role in other systems as it is expressed in many other embryonic tissues [18-20] and in epithelial cells [19,20]. It is furthermore overexpressed in endometrioid carcinoma [21] and down-regulated in gastric cancer [22]. The *in vivo *function of RUNX1 is therefore yet to be fully understood.

Here we describe the combination of a number of genomic and bioinformatic approaches to identify biological pathways downstream of RUNX1, and report on a number of processes in which RUNX1 is likely to be involved. We also took advantage of the integration of these approaches in order to identify novel RUNX1 target genes.

## Results

### Gene expression profiling of cells harboring different levels of RUNX1

**Figure 1:**
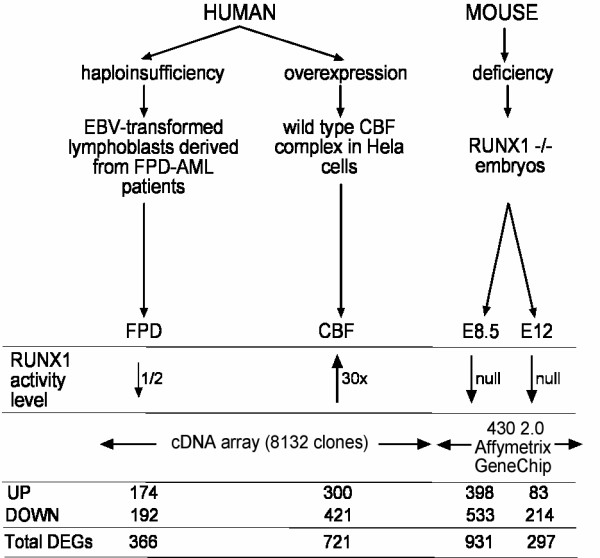
**Gene expression profiles and overlaps**. The three platforms used in this study are indicated. The number of up-, down- or all differentially expressed genes (DEGs) are indicated below each platform.

Three different model systems were used to identify the biological pathways regulated by the RUNX1 transcription factor. These were haploinsufficiency using FPD-AML patient B cell lines (FPD), overexpression of CBF complex (CBF) in HeLa cells and Runx1 deficiency in mouse embryos (E8.5 and E12) (Figure 1).

Lymphoblastic cells derived from FPD patients heterozygous for a RUNX1 frameshift mutation (R135fs) were first analyzed. This mutation results in haploinsufficiency of RUNX1, as the mutant protein has lost its capacity to bind DNA and to transactivate the expression of the target genes [9]. Quantitative RT-PCR on these non-leukemic lymphoblastic cells showed that affected individuals express approximately 55% of the transcript level observed in unaffected individuals (see Additional File 1 :Figure S1). The genes differentially expressed between two affected and two non-affected cell lines are therefore largely the result of a low dosage of RUNX1 protein. Using human cDNA microarrays with the Hs8k cDNA clone library from Research Genetics and a selection of control spots, 366 genes were identified as differentially expressed, of which 52% (192/366) were down-regulated in affected individuals (Figure 1 and see Additional File 2).

For overexpression studies, HeLa epithelial cells were transduced using adenoviral vectors. FACS analysis showed that over 90% of HeLa cells were transduced by a EGFP-expressing adenovirus (data not shown). This system results in a highly homogenous cell population in which small changes of expression can be identified. The wild type CBF complex α-subunit, RUNX1, was overexpressed together with the β-subunit, CBFβ (see Additional File 1: Figure S2) and seven hybridizations were performed. Following overexpression of the CBF complex, 721 genes were differentially expressed including the up-regulation of 42% of the genes (300/721; Figure 1 and see Additional File 2).

Finally, we compared the expression profiles of two wild type and two *Runx1 *knockout mouse embryo propers at each embryonic stages E8.5 and E12 using Affymetrix chips. Despite the heterogeneity of the samples, 931 and 297 genes were differentially expressed at embryonic stages E8.5 and E12, respectively. Of these genes, 57% (533/931) and 72% (214/297) were down-regulated in the knockout embryos (Figure 1 and see Additional File 3). These differences in expression are likely to reflect the lack of hematopoiesis and the premature death, respectively, observed in the Runx1 embryos.

We then compared the different datasets using a mean-rank gene set enrichment test (MR-GSE) in order to determine the level of connection between the 3 approaches (FPD cell lines, CBF overexpression and *Runx1 *knockout mouse embryos), disregarding the cell type and the organism. High correspondence was observed between the two human datasets. The correspondence between the human and the mouse datasets was not as good, although still significant. This might partially be explained by the difficulties of matching human and mouse platforms (see Additional File 1: Figure S3).

### Correlation with clinical AML samples

**Figure 2:**
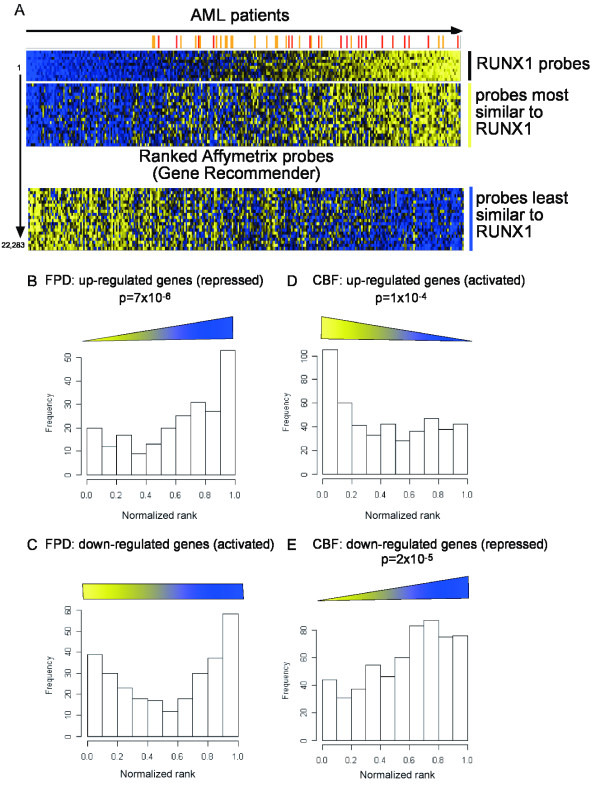
**Correlation with clinical AML data**. A. Published microarray data on 285 AML patients [23] were ordered using Gene Recommender according to the expression pattern of the 11 probe sets for RUNX1. The patients with t(8;21) are marked in orange and those with inv(16) in red. Probes co-regulated with RUNX1 are highly ranked (yellow bar), whereas probes showing an expression pattern the least similar to RUNX1 are ranked lowest (blue bar). B-C. Random permutations were performed to compare the rank of the genes differentially expressed in FPD platform and random set of genes. The histograms show the percentage of up- or down-regulated genes in FPD relative to their rank with "0" being the probes co-regulated with RUNX1 (yellow) and "1" being the probes the least similar to RUNX1 (blue). The trends observed in the histograms are represented as triangles or rectangle. D-E. Similar histograms showing percentage of up- or down-regulated genes in CBF relative to their rank.

It was first necessary to determine whether the genes identified in nonmyeloid cells in this study may play a role in myeloid leukemia development. We therefore compared our data to previously published microarray data obtained from 285 AML and 8 healthy samples [23], using the MR-GSE test. The high correspondence between the FPD-AML and CBF datasets had already suggested that a large number of downstream genes were similar between epithelial and lymphocytic cells. Therefore we used each approach as representative of the *RUNX1 *gene dosage, regardless of the cell type. The AML samples used in the comparison include 22 patients with a t(8;21) translocation, which fuses *RUNX1 *to *ETO*, and 18 patients with inv(16), which fuses the co-factor *CBFβ *to *MYH11*. The other samples include a range of common alterations or no identified mutations. RUNX1 activation targets should be positively correlated with RUNX1 expression whereas repression targets should be negatively correlated. Therefore we ranked all the probes-sets on the microarrays according to their correlation with RUNX1 across the 293 AML and normal samples (Figure 2A). MR-GSE tests demonstrated that genes up-regulated in the FPD-AML patients (likely to represent genes repressed by RUNX1), had an expression trend opposite to RUNX1 in the AML patients, suggesting indeed that these genes are repressed *in vivo *in the presence of RUNX1 (p = 7 × 10^-6^; Figure 2B). On the other hand, the down-regulated genes do not show any statistically significant trend (Figure 2C). Similarly, the genes activated by the exogenous CBF complex had an expression pattern similar to RUNX1 across the clinical samples (p = 1 × 10^-4^; Figure 2D), whereas genes repressed by the CBF complex had an expression pattern opposite to RUNX1 (p = 2 × 10^-5^; Figure 2E).

MR-GSE tests also showed that genes differentially expressed in the B cell lines derived from FPD-AML patients tended to be differentially expressed in the blasts and mononuclear cells of 22 clinical patients with a t(8;21) translocation (p = 10^-10^) and of 18 patients with the inv(16) abnormality (p = 3.5 × 10^-9^). For example, the top 14 differentially expressed genes in the FPD-AML dataset that are also differentially expressed in the clinical samples are shown in Additional File 1 (Table S3). As a whole, these results demonstrate that the genes identified in our study are likely to play an important role in the development of the disease.

### Biological processes regulated by RUNX1: bioinformatic approaches

**Figure 3:**
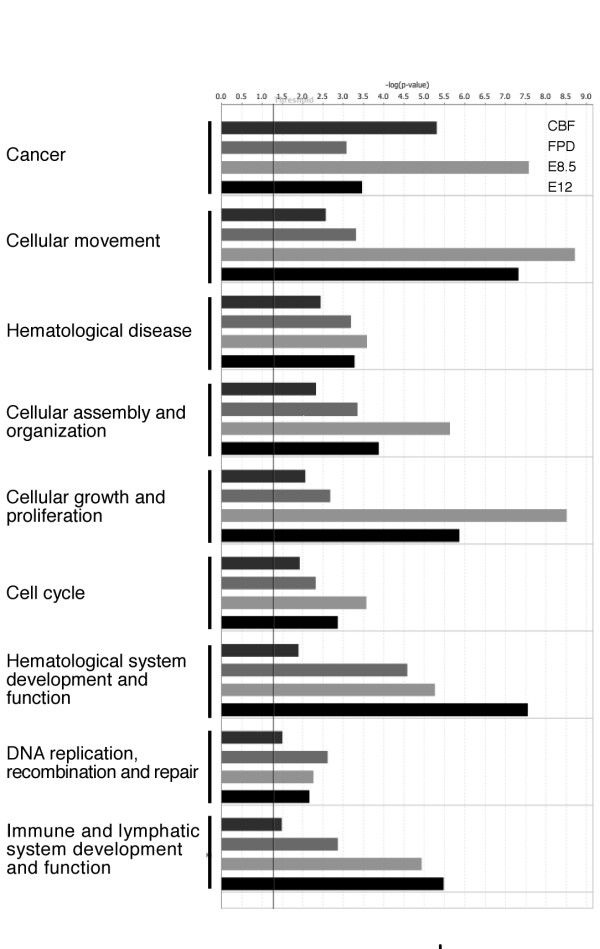
**Processes identified by Ingenuity Pathways Analysis**. Evidence that each dataset is involved in the given function as determined by the use of Ingenuity Pathways Analysis (Ingenuity Systems, ). The threshold for the significance is indicated by a vertical bar and represents a p-value of 0.05.

**Figure 4:**
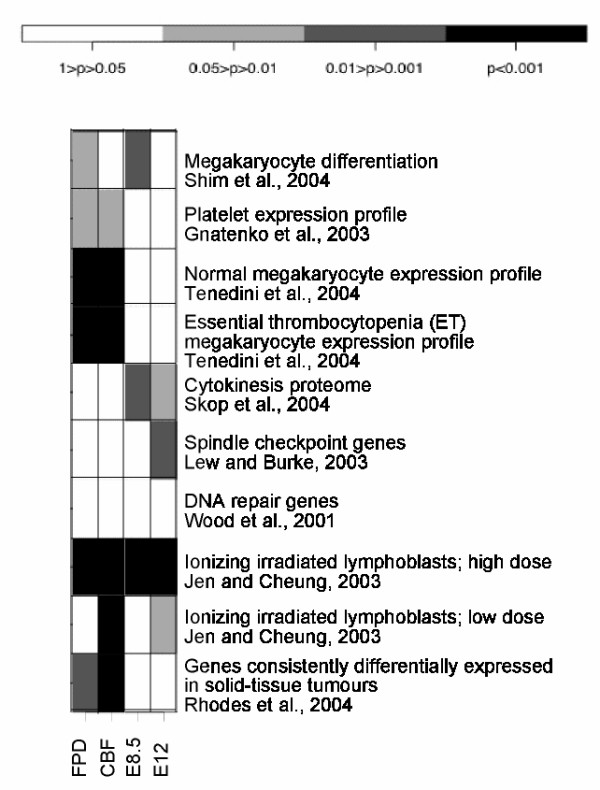
**MR-GSE test**. Representation of the p-values (corrected for multiple testing) resulting from the MR-GSE test for each dataset and 10 gene sets specified in Additional File 1 (Table S4). In brief they are gene sets Mekagaryocyte differentiation, Identification of genes involved in the differentiation of megakaryocytes. DEGs between stem cells and differentiated megakaryocytes; Platelets, Transcription profiling of human blood platelet; ; Normal megakaryocytes, Genes highly expressed in megakaryocytes; ET megakaryocytes, Genes highly expressed in essential thrombocytopenia megakaryocytes; Cytokinesis proteome, Identification of proteins present in the midbody during cytokinesis; Spindle checkpoint, Review ; DNA repair, Review; Lymphoblast irradiation; high dose, Effect of ionising radiation on lymphoblasts; Lymphoblast irradiation; low dose, Effect of ionising radiation on lymphoblasts; Genes DE in cancer, Meta-analysis of cancer microarray data to identify genes consistently DE in tumours. This represents whether the genes present in the published gene sets are also differentially expressed in our expression profiles. For example, the genes expressed in normal or diseased megakaryocytes (lines 3 and 4) are significantly represented in the differentially expressed genes identified in the FPD and CBF approaches.

Bioinformatics tools taking into account all differentially expressed genes (direct and indirect RUNX1 targets) were used to systematically identify the biological processes in which RUNX1 may be involved. A number of gene ontology (GO) annotations were significantly enriched in each dataset (Table 1). Some were identified in more than one dataset such as "cadmium ion binding" and "immune response". Other significantly represented processes were identified through the use of Ingenuity Pathways Analysis (Ingenuity Systems, ) (Figure 3). These include cancer related genes as well as genes involved in hematological disorders. To complete this analysis, a MR-GSE was also performed using a number of published gene sets related to thrombocytopenia, leukemia and cancer (Figure 4, see Additional File 1: Table S4 and Additional File 4). Significant correlation was obtained between the microarray datasets and a number of these sets of genes, including genes involved in megakaryopoiesis and cytokinesis, genes differentially expressed following irradiation of lymphoblasts, and genes consistently differentially expressed in solid-tissue tumors.

**Table 1:** Gene ontology enrichment

	FPD	CBF	E8.5	E12
GO: Biological processes	**Immune response p = 6.5 × 10^-5 ^36 genes**	Macromolecular complex assembly p = 0.02 47 genes	Blood vessel development p = 0.06 15 genes	Response to external stimulus* p = 0.0003 18 genes
	Negative regulation of apoptosis p = 0.002 16 genes	Cell growth p = 0.02 21 genes		Behavior p = 0.0003 14 genes
	Response to biotic stimulus p = 0.002 19 genes			**Immune system process p = 0.0006 18 genes**
	Cell proliferation p = 0.01 36 genes			

GO: Molecular functions	**Cadmium ion binding p = 0.002 4 genes**	RNA binding p = 0.03 50 genes		IgG binding p = 0.006 3 genes
		**Cadmium ion binding p = 0.03 4 genes**		Ferric-chelate reductase activity p = 0.03 2 genes
				Polysaccharide binding p = 0.03 6 genes

GO: cellular component		Spindle p = 0.06 11 genes	Cell junction p = 0.06 14 genes	Cell surface p = 0.05 9 genes
				Extracellular space p = 0.06 37 genes

InterPro motifs (FatiGo)	**Vertebrate metallothionein p = 0.0001**	**Vertebrate metallothionein p = 0.02**		
		Tubulin p = 0.04		

The most significant gene ontology annotations are indicated for each dataset as identified through GOStat in April 2007. InterPro motifs were identified through the FatiGo program. The p-values are corrected for multiple testing (False discovery rate, Benjamini).

### Biological processes regulated by RUNX1: *in vivo *confirmations

We designed a series of assays that were performed on either cell lines, or directly on samples from FPD-AML patients with RUNX1 mutations, to confirm the disturbance of several interesting biological processes identified by the above approaches.

### Heterozygous *RUNX1 *point mutations affect proliferation

**Figure 5:**
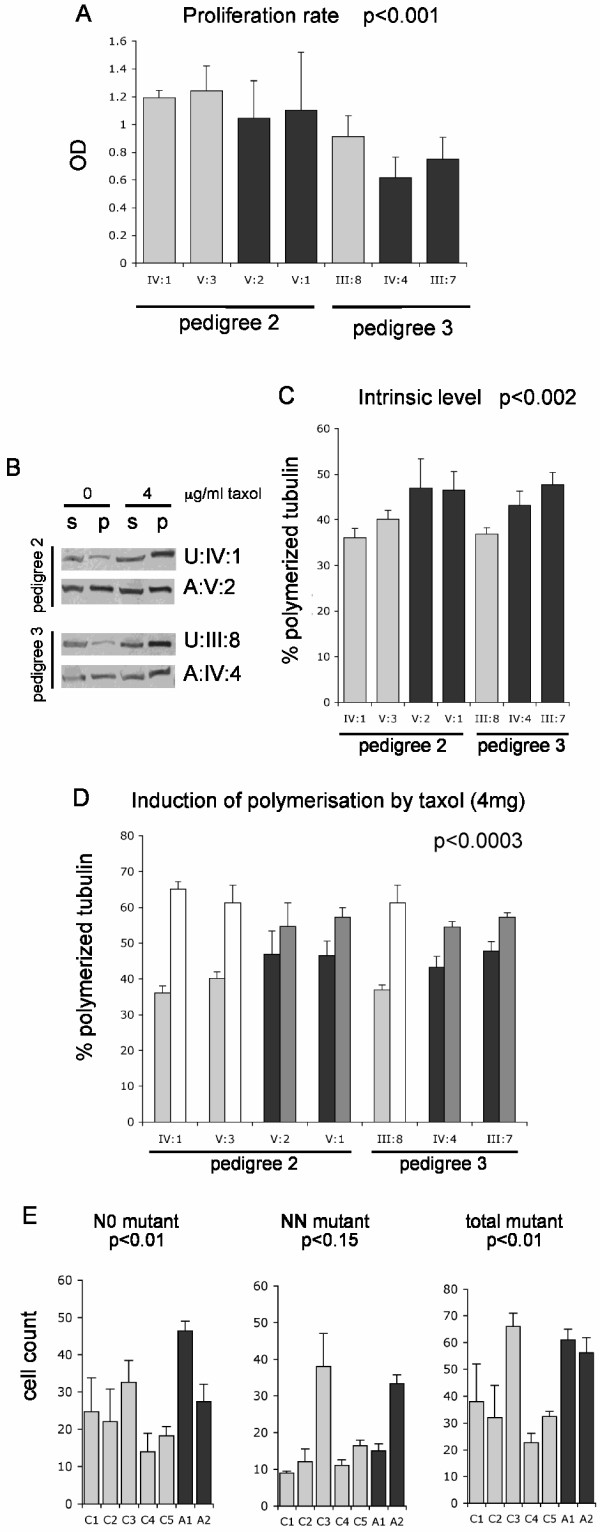
**Functional assays on FPD-AML cell lines**. A. The results of a BrdU proliferation assay are indicated for each cell line. Dark bars indicate affected individuals. The standard errors of two independent replicates are shown. A two-way ANOVA resulted in a significant p-value (p < 0.001) between affected and unaffected individuals. B. Examples of the tubulin polymerization assay for an affected and an unaffected individuals in each family. s:soluble tubulin; p:polymerized tubulin. C. The percentage of polymerized tubulin is shown for each cell line. Dark bars indicate affected individuals. The standard errors of three independent replicates are indicated. A two-way ANOVA resulted in a significant p-value (p < 0.002) between affected and unaffected individuals. D. Percentage of polymerized tubulin in the same cell lines before (darker left bars) and after (second bars) induction of polymerization by Taxol. A significant smaller induction is observed in affected individuals (dark bars) as demonstrated by an ANOVA (p < 0.0003). E. Glycophorin A assay. The numbers of N0 (loss of the M allele), NN (mutation changing M to N allele) or total mutant (both N0 and NN) cells are indicated for each individual. The standard errors of three to five technical replicates are indicated. Dark bars represent affected individuals (A1-A2). The control C5 is the unaffected sister of patient A1. ANOVAs were performed for each kind of mutation and the p-values are indicated.

RUNX1 is thought to be involved in the balance between cell proliferation and differentiation, whose disruption leads to leukemia development. However, the molecular mechanisms behind this regulation are not known. We observed that genes participating in cellular proliferation were significantly enriched in both FPD and CBF datasets (Table 1 and Figure 3). The genes responsible for this enrichment are indicated in Additional File 1 (Table S5). We therefore performed a BrdU proliferation assay in order to determine whether a subtle proliferation defect was present when RUNX1 level was lower in FPD-AML patients. A slower proliferation was indeed observed in FPD-AML lymphoblasts derived from two independent families compared to unaffected cells (Figure 5A, p < 0.001).

### RUNX1 modulates microtubule stability

A significant enrichment of molecules containing a common tubulin motif was observed following overexpression of the CBF complex (Table 1). Five tubulin isoforms were down-regulated following overexpression of the CBF complex. These data led to the observation that CBF overexpression affected the expression of 57 genes associated with cytoskeletal structures according to GO annotation (see Additional File 1: Table S6). This class of genes was not significantly represented in the dataset from the FPD-AML cell lines, however this may be the result of the not complete knock-down of RUNX1 in the affected individuals leading to small changes that are not detected by microarray analysis. Therefore we also tested whether microtubule stability was affected in these cell lines. Significantly higher microtubule polymer levels were observed in the affected patients compared to the unaffected individuals (Figure 5B and 5C; p < 0.002). Furthermore, the microtubules in affected cells could not be stabilized using the drug Taxol to the same extent as the unaffected cells (Figure 5D; p < 0.0003). This might result from the inability of the drug to bind to the microtubule molecule because of the unusual presence of other microtubule stabilizing proteins or from a lack of soluble tubulin molecules in the cellular environment. In any case, these results suggest that RUNX1 is involved in microtubule dynamics.

Neither the proliferation nor the tubulin defects are due to the EBV transformation of the cell lines as many independent proliferation and tubulin polymerization assays performed on lymphoblastic cell lines derived from families with predispositions to various haematological malignancies do not show similar familial clustering (data not shown).

### Genomic instability

Highly significant correspondence was observed between the FPD, CBF and mouse datasets and the genes switched on after irradiation of lymphoblasts (Figure 4). We used a glycophorin A assay to test whether the FPD-AML patients are more prone to somatic genetic mutations than unaffected individuals. This test assesses the frequency of mutation events occurring at the glycophorin A locus in erythroid progenitors in blood of heterozygous individuals (MN phenotype) [24]. Although more samples would be necessary for corroboration, a significant trend was present between the blood of two affected patients and five unaffected individuals, suggesting that a subtle increase of mutation rate may occur when RUNX1 activity is impaired (Figure 5E; p < 0.01). This increased mutation rate appears to be higher in the assay that would detect deletions (NO), that are the predominate mutations arising due to ionizing irradiation [25].

### Identification of potential novel RUNX1 target genes – co-expression in human tissues and hematopoietic cell lines

We reasoned that direct RUNX1 target genes must be expressed in the same tissues or cells as RUNX1. Thus, the expression patterns of a number of differentially expressed genes, chosen due to potential functions in leukemia development, were compared to that of RUNX1 (see Additional File 5). The expression of 22 genes in 20 human tissues, 19 hematopoietic cell lines and normal human bone cells was assessed using cDNA panels [26]. 9 of these genes show a high expression in a number of hematopoietic cell lines and all the others show common expression with RUNX1 in various tissues such as liver and peripheral blood leukocytes (PBLs).

### Identification of potential novel RUNX1 target genes – data overlaps

**Figure 6:**
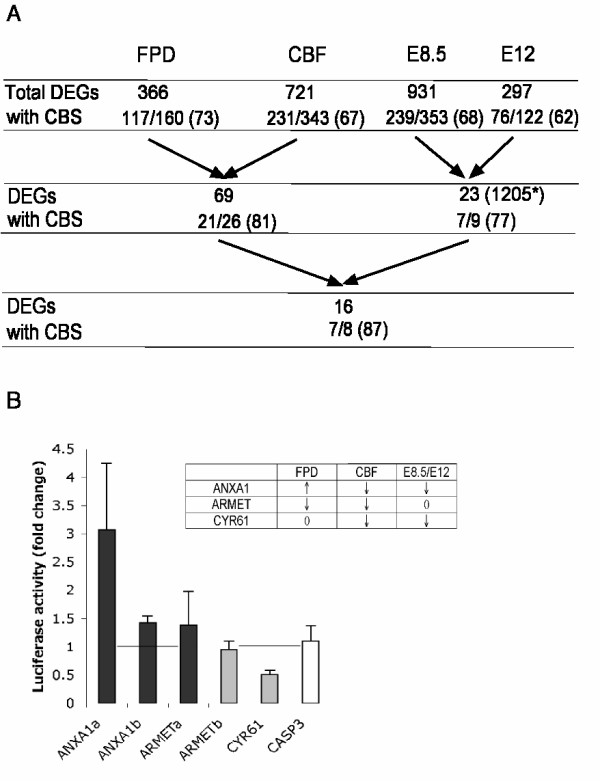
**A. Overlaps between the datasets and percentage of genes with a RUNX1 binding site in their regulatory regions**. The overlaps between the different platforms are represented with arrows. * indicates that the genes differentially expressed in at least one of the mouse datasets are considered for the following overlap. The number of differentially expressed genes (DEGs) containing a conserved RUNX1 binding site (with CBS) in their regulatory regions, as determined by the oPOSSUM program [27], over the number of analyzed genes is indicated for each dataset and overlap. The corresponding percentage is indicated in brackets. B. Luciferase assay for 5 RUNX1 binding sites corresponding to 3 differentially expressed genes. The transactivation activity of RUNX1 over these sites was measured as the fold change of the luciferase activity in the presence of the CBF complex compared to the endogenous activity of each construct. The standard errors of three independent replicates are shown. CASP3 was shown as a negative control as no binding site was found for this gene. The difference in expression for the three genes in each dataset is indicated in the table. 0 means no difference in expression, ↓ stands for down-regulated and ↑ stands for up-regulated.

In order to distinguish between the direct RUNX1 target genes and those effected further downstream by a disregulation of RUNX1 level, we hypothesized that the genes in common in more than one dataset were more likely to be at the top of the genetic pathways regulated by RUNX1 and to be enriched for direct target genes. As suggested by the significant MR-GSE results, we observed statistically significant overlap between each dataset. Among the 366 genes differentially expressed in FPD-AML cell lines, 69 genes were also differentially expressed following overexpression of the CBF complex, while only 32 were expected by chance (Figure 6A). As anticipated when comparing an under- and overexpression system, 61% (42/69) of the genes in this overlap were differentially expressed in the opposite direction. Among these 69 genes 16 were also differentially expressed in at least one embryonic stage of the *Runx1 *knockout embryos (Table 2, Figure 6A).

**Table 2:** Genes differentially expressed in FPD, CBF and in E8.5/E12

**Gene name**	**RefSeq**	**RUNX1 BS**
ITM2C	NM_030926	y
GLO1	NM_006708	ND
OGT	NM_003605	y
ALAS1	NM_000688	ND
HSPA4L	NM_014278	y
PPIB	NM_000942	ND
CIB1	NM_006384	N
BASP1	NM_006317	y
TACC1	NM_006283	ND
CTSC	NM_001814	y
PBX3	NM_006195	y
TGFBR3	NM_003243	ND
ANZA1	NM_000700	y
ELF1	NM_172373	ND
IFRD1	NM_001550	ND
MT1G	NM_005950	ND

RUNX1 BS: presence of a RUNX1 binding site in the regulatory region of the gene as determined by oPOSSUM. y stands for the presence of binding site and ND stands for not determined due to the absence of the gene in oPOSSUM.

### Identification of potential novel RUNX1 target genes – regulatory region analysis

In order to accumulate evidence that some of the genes present in these overlaps are direct target genes, we searched for human RUNX1 binding sites, which were conserved in mouse using the oPOSSUM software ( see Additional File 1) [27]. Many differentially expressed genes contained at least one conserved RUNX1 binding site in their regulatory regions and the overlaps between the datasets show a higher enrichment for such genes as hypothesized above (Figure 6A).

The regions flanking five putative conserved binding sites identified in three differentially expressed genes, and one negative control region, were cloned upstream of a luciferase reporter gene and co-transfected together with plasmids expressing RUNX1 and CBFβ. These genes were selected because of their presence in the overlap between the human datasets and/or their interesting functions; ANXA1 (Annexin 1) is involved in cell proliferation and cytoskeleton regulation; ARMET (Arginine-rich, mutated in early stage tumors) is mutated in cancer; CYR61 (Cysteine-rich, angiogenic inducer, 61) promotes proliferation and angiogenesis. An increase in luciferase activity was observed for ANXA1 binding sites and for one of the ARMET binding sites and a diminution of the luciferase activity was observed for the CYR61 binding site (Figure 6B). No modification of the luciferase activity was observed for a sequence derived from the negative control CASP3 regulatory region (where no conserved binding site was identified by the oPOSSUM program). It is likely that a combination of a number of binding sites and the presence of additional co-factors are necessary for a correct and synergistic *in vivo *regulation of these genes and it might explain the small activity observed for the ARMET binding sites. It might also explain the activation of the ANXA1 site while this gene was repressed by the overexpression of the CBF complex.

## Discussion

**Figure 7:**
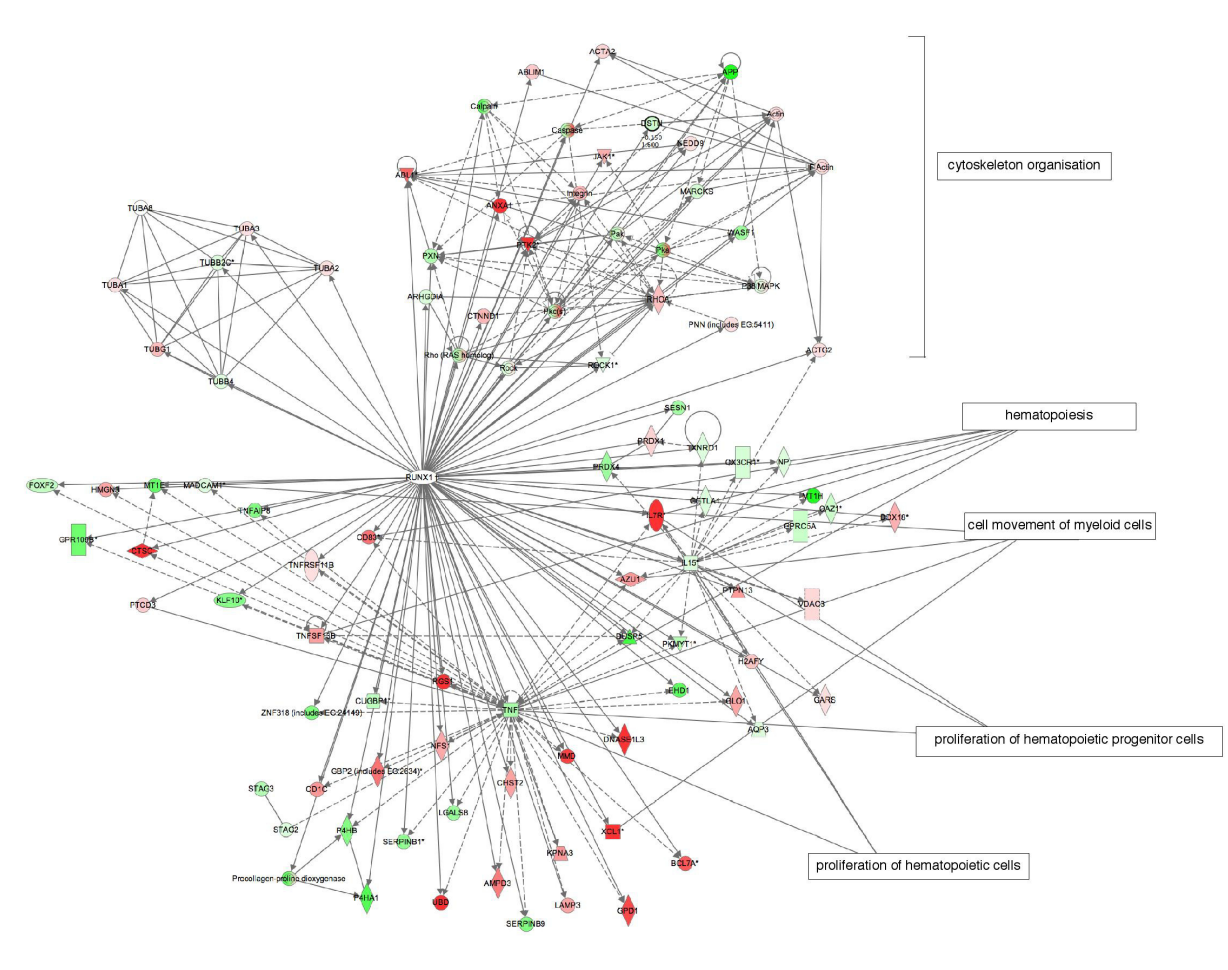
**Part of the networks downsteam of RUNX1**. Additional data from the literature and our studies were used to update the standard Ingenuity Pathway System (Ingenuity^® ^Systems, ) network analyses. Genes up-regulated (red) or down-regulated (green) in either FPD or CBF are indicated. Selected chosen functions with significant network nodes are shown including all the genes involved in cytoskeleton organization. Grey arrows represent transcriptional regulation, grey lines represent direct interaction, dotted lines represent indirect link. Each kind of molecule is represented by a different symbol (see ).

*RUNX1 *is one of the most frequent targets of somatic mutations in leukemia and is mutated in an autosomal dominant disorder affecting platelets and predisposing to leukemia development. Better characterization of its *in vivo *function is likely to give insight into the mechanisms leading to the development of leukemia, and will provide new candidate genes for leukemogenesis. We do not believe that as a transcription factor and master regulator of hematological cancers, RUNX1 will alter the function of only one oncogenic molecule, but multiple molecules in the same pathways, and our analyses and functional assays are carefully designed to study these effects. We have described a combination of genomic and bioinformatic approaches to identify the biological pathways and genes regulated by RUNX1, an overview of which is in Figure 7. Each approach independently provides a large source of data to identify RUNX1 targets according to *RUNX1 *gene dosage. However, the combination of them is powerful because of their convergence. Although the approaches described here are not the ideal models to study myeloid leukemia, each of them has their own advantages and their integration compensates for their limitations: 1) The use of cells derived from patients harbouring a *RUNX1 *mutation but who have not yet developed leukemia allow us to observe effects, largely due to changes in RUNX1 dosage. However, it should be kept in mind that due to the difficulties of obtaining myeloid cell lines, these studies were performed in lymphoid cells. 2) The overexpression system using HeLa cells provided a highly homogenous cell population, which is necessary to perform gene expression profiling. 3) The knockout mouse embryos represent various cell types, however they give us global information of the complete absence of RUNX1, which is difficult to obtain using cell lines. Efficient and homogenous knockdown levels are indeed difficult to obtain using siRNA especially in hematopoietic cells [28].

The highly significant correlation observed between the genes identified in the FPD-AML cells and the overexpression system and clinical data on AML samples supports the hypothesis that large number of genes would be broadly regulated by RUNX1 in our various approaches disregarding of the cell type. Genes identified as differentially expressed following disregulation of RUNX1 expression level and/or in these AML samples are good candidates for targets of secondary hits during leukemogenesis downstream of RUNX1 mutation. The various approaches described in this study, including conserved binding sites and co-expression studies, will also help to further prioritize genes that might sustain secondary hits. For example, the gene encoding the Cyclin D3 (CCND3) was differentially expressed following overexpression of the CBF complex and mutations in this gene have been described in acute myeloid leukemia patients [29].

In order to generate insights into the *in vivo *role of RUNX1, we employed bioinformatics tools to identify processes that were changed following alteration of RUNX1 expression level. We have shown that genes involved in megakaryopoiesis tend to be differentially expressed in the FPD and CBF datasets, demonstrating that a large number of the differentially expressed genes may play a role in platelet formation. Enrichment for genes involved in cell proliferation was also observed in both the FPD and CBF datasets, and functional assays on the FPD-AML cell lines showed that heterozygous mutation of *RUNX1 *reduced proliferation of lymphoblasts. These data validate our integrative approach as they confirm studies in transgenic mice expressing the fusion proteins CBFβ-MYH11 [30] and RUNX1-ETO [13], which both act in a dominant negative fashion over the wild-type protein. These mice show a decrease in both lymphoid and myeloid cell proliferation. This observation also correlates with mouse data showing that Runx1 promotes cell cycle progression from G1 to S phase [31]. An anti-proliferative effect of a RUNX1 mutant protein may have an oncogenic effect due to an improper balance between proliferation and differentiation. For example, overexpression of RUNX1 usually results in ALL while complete or partial loss of RUNX1 results in AML development.

Our integrative approach unraveled a novel process that may play an important role in RUNX1 function, involving the cytoskeletal dynamics. Indeed following the finding that an enrichment of microtubule and cytoskeleton related molecules was observed when the CBF complex was overexpressed, functional assay using the FPD-AML cells demonstrated an increase of polymerized microtubules in FPD-AML affected cells compared to cells from unaffected individuals. Microtubules are important in many processes such as cell migration, cell division, cellular transport and signal transduction [32] and microtubule remodeling is essential during the cell cycle, especially during mitosis when a correct microtubule network is essential for proper chromosomal segregation [33]. Interestingly, the fusion protein, CBFβ-MYH11 that results from inv(16), co-localizes with the actin cytoskeleton and disorganizes stress fibers and F-actin structures [34]. A mild microtubule defect might partially explain the platelet defect observed in FPD-AML patients, as microtubules are necessary at several different stages of megakaryopoiesis including endomitosis, production of platelets from mature polyploid megakaryocytes, and release of the content of platelet granules [35]. Moreover, mutations in the actin-binding protein WASP and the myosin heavy chain MYH9 cause the Wiskott-Aldrich [36] and May-Hegglin [37] syndromes of thrombocytopenia, respectively. However, RUNX1 is likely to regulate only specific tubulin isoforms or tissue-specific cytoskeleton-associated proteins as a strong cytoskeleton defect would be more detrimental to the whole organism. In addition, the dosage of normal RUNX1 activity necessary for normal function might differ according to cell type, and some cell types may be more susceptible than others to perturbation in RUNX1 levels. Interestingly, Taxol resistant leukemic cells have been shown to have a reduced total level of tubulin and an increased level of polymerized tubulin [38], similar to the results seen in the FPD-AML cells. Furthermore, a high level of survivin (BIRC5), which was down-regulated following overexpression of the CBF complex, is associated with resistance to Taxol [39]. This is the first evidence demonstrating a relationship between RUNX1 and microtubule dynamics.

Finally, we showed that the predisposition of FPD-AML to develop leukemia may be due to an increased rate of mutation in *RUNX1 *heterozygous cells. Every dataset showed significant correspondence with genes involved in DNA damage response. Although not conclusive, the glycophorin A assay, which measures the frequency of the progeny of mutated erythrocyte precursors in blood, showed a mild increase in mutation frequency in FPD-AML patients compared to unaffected individuals. Recently, it was shown that the RUNX1-ETO fusion protein induces mutations in transfected U937 myeloid cells [40]. This study demonstrated that the fusion protein regulates many genes involved in the base excision repair pathway, which mainly corrects for point mutations. Furthermore, a higher incidence of leukemia in CBFβ-MYH11 chimeras compared to normal chimeras when exposed to ENU mutagenesis has also been observed [41,42]. This demonstrates that alteration of RUNX1 function may increase the rate of mutation and lead to an accumulation of mutated cells.

The three processes described here (proliferation, cytoskeleton stability and genomic instability) are tightly interconnected and may explain the phenotype observed in FDP-AML patients. Indeed, a proliferation defect would have an impact on megakaryopoiesis and cytoskeleton remodeling. In turn, a cytoskeleton defect could also affect proliferation and trigger chromosomal aberrations. The necessary threshold level of RUNX1 expression is likely to be cell-specific, explaining why *RUNX1 *heterozygous mutation affects only hematopoietic cells; nevertheless, our observations could conceivably suggest possible involvement of RUNX1 in solid-tissue tumor.

We also identified new potential RUNX1 target genes by analyzing the regulatory regions and the expression pattern of the differentially expressed genes present in the overlaps between the different platforms. Many RUNX1 target genes have already been described in the literature, mainly from *in vitro *studies and in mouse cells [43,44]. Four of the published target genes, CSF1R, MYB, MPO and TIMP1, were differentially expressed in the *Runx1 *knockout embryos. In addition, target genes that were described more recently, including CCND3 [45] and IGFBP3 [46], were identified following overexpression of the CBF complex. That there was not more correlation may be due to incomplete microarray platforms, but more importantly is likely to reflect the bias present in the published RUNX1 target genes that were identified because of their primary role in hematopoiesis and these may not represent the most common RUNX1 target genes. Interesing candidates were among the 16 genes differentially expressed in every dataset, such as Annexin I (ANXA1), which was shown to reduce inflammation, by inhibiting neutrophil recruitment [47] and has an anti-proliferative effect by inducing aberrant cytoskeleton formation [48]. This gene is likely to play an important role downstream of RUNX1.

## Conclusion

In summary, this combination of gene expression profiling platforms allowed prioritization of novel candidate genes for leukemogenesis according to distinct parameters and has shed light on RUNX1 functions by identifying biological pathways downstream of RUNX1 such as microtubule stability and genomic instability and identified a large number of potential novel RUNX1 target genes. Whether or not these are direct RUNX1 targets remains to be demonstrated by further research.

## Methods

### Adenovirus production

Recombinant adenoviruses expressing *RUNX1 *p49 isoform [49] or CBFβ were generated as described [50], except that VmRL-CMV1 and pSCOT were used as the adenovirus backbone and transfer vector respectively. For details, see Additional File 1.

### Cell lines and RNA extraction

EBV-transformed lymphoblasts generating B cell lines from FPD-AML patients (Pedigree 2, individuals V:1 and V:2;) [9] and related unaffected individuals (Pedigree 2, individuals IV:1 and V:3) were used for the FPD microarray dataset. HeLa cells (4 × 10^7^) were infected with a multiplicity of infection (MOI) of 100 for each adenovirus and incubated for 48 hours. The Qiagen RNeasy maxikit was used for the extraction of total RNA in each case. *Runx1 *knockout and wild-type embryo propers at embryonic stages E8.5 and E12 were homogenized in Trizol (Invitrogen) and total RNA extracted following the manufacturer's protocol.

### Mouse samples

Runx1 knockout mice have been previously described [16]. They are maintain on a BalbC genetic background at the Biological Resource Center, (Biopolis, Singapore) and all animal experiments followed the guidelines set by the National Advisory Committee for Laboratory Animal Research. Wild-type and Runx1 knockout mouse embryo propers were harvested at embryonic stages E8.5 and E12.

### cDNA Microarray hybridization

cDNA microarrays were printed by the Australian Genome Research Facility (AGRF) with the Hs8k cDNA clone library from Research Genetics and a selection of control spots. In total there were 8132 EST probes printed in duplicate. The array also contained 12 copies of the Lucidea Universal ScoreCard controls (Amersham). Labeling, hybridization, and washing were performed as described [51]. In the case of the FPD dataset, four hybridizations were performed comparing two affected individuals against two unaffected individuals of pedigree 2. For the overexpression system, 2 different RNA samples from HeLa cells overexpressing EGFP were used as reference and 2 different RNA samples from HeLa cells overexpressing RUNX1 and CBFβ were used as experimental RNAs. Seven hybridizations (including 3 dyeswaps) were performed. The data were filtered for genes whose difference in expression was due to EGFP, using four hybridizations between EGFP expressing cells and normal HeLa cells.

### Affymetrix genechip hybridization

Labelling, hybridization and washing were performed by the AGRF following the Affymetrix protocol (701725 rev5). Briefly, total RNA (100 ng) was amplified using T7-oligo dT and the Megascript T7 kit (Ambion). A second round of cDNA synthesis was performed using the total amount of the amplified RNA. Biotin-labeled RNA was subsequently synthesized using the GeneChip IVT Labeling Kit. Labelled RNA (15 μg) was fragmented and the mouse genome 430 2.0 arrays were hybridized overnight and washed as described before being scanned using a GeneChip scanner 3000 (Affymetrix). Two biological replicates were used for each condition.

### Microarray analysis

The cDNA microarray images were analyzed using SPOT software [52]. Spots were assigned quality weights based on their segmented pixel areas and the log-ratios were print-tip loess normalized [53]. Duplicate printings of each probe on each array were combined using the common correlation method of [51]. For the mouse Affymetrix GeneChips, the intensities for each probe set were normalized and summarized using the Robust Multi-array Analysis algorithm [54]. Differential expression was assessed using empirical Bayes moderated t- and F-statistics from the LIMMA package [55]. Recognizing that p-value calculations make normality and other distributional assumptions, which are hard to verify for microarray data, we decided to use control probes and appropriate plots to guide our criteria for differential expression as far as possible. For the cDNA data, conservative threshold values for differential expression were chosen to minimize the false-positive and false-negative rates estimated from Scorecard control probes printed on the arrays. This resulted in a threshold value of |t|>4 for the FPD data. Of 204 calibration control probes printed on the arrays, none reached this cutoff for statistical significance, suggesting a false discovery rate less than 1/204, without relying on any distributional assumptions. For the mouse Affymetrix data, a threshold of |t|>3 was chosen from a q-q plot of the moderated t-statistics.

For the overexpression system arrays, a combination of criteria was used to assess differential expression. These arrays were analyzed as part of a larger microarray study using the same overexpression system to study a range of AML related genes. Genes with |t|>4 were initially assigned as differentially expression, with only one calibration control probe reaching this threshold. A series of nested F-tests (with p-value cutoff 1e-5) was also performed using the larger dataset in order to get an improved estimate of the number of genes significantly differentially expressed in more than one condition simultaneously. This increased the number of differentially expressed genes by a third. Finally, genes were removed from the differentially expressed list if their response to *RUNX1/CBFβ *transduction was not significantly greater than their response to the adenovirus alone.

All the analyzed datasets have been deposited at the NCBI Gene Expression Omnibus  under accession numbers GSE2592 (mouse Affymetrix data), GSE2593 (overexpression experiment) and GSE2594 (FPD-AML arrays).

### Mean-rank gene set enrichment tests (MR-GSE)

A version of statistical gene set testing was used to investigate associations between the expression profiles obtained from different experiments. Each test uses a set of genes selected as differentially expressed in one data set (the reference dataset) and determines whether the gene set tends to be highly ranked in another dataset (the test dataset). The test statistic is the mean rank of the gene set in the test dataset. This approach, which we call mean-rank gene set enrichment (MR-GSE), is very similar to Tian et al's T_k _test [56] and Kim and Volsky's PAGE test [57]. The main difference is that MR-GSE averages the ranks of t-statistics instead of t-statistics themselves, which makes it less influenced by individual genes in the gene set. This has the advantage of giving more weight to gene sets with a larger number of active genes, and it also allows us to use the same testing procedure with a range of ranking procedures other than t-statistics. Where possible, MR-GSE is used with moderated t-statistics rather than ordinary t-statistics, as these are preferable for microarray analysis including gene set testing [56,58]. Unlike earlier Gene Set Enrichment Analysis methods [59], MR-GSE can be used to test individual gene sets in isolation and has good power even for microarray experiments with small to moderate sample sizes.

The null hypothesis tested by MR-GSE is that the gene set is randomly chosen. When the reference and test datasets share the same microarray platform, p-values can be computed using Wilcoxon two-sample rank tests [60]. When the reference and test datasets are based on different microarray platforms (cDNA vs Affymetrix), the p-values were instead computed using random permutations of probes on the reference arrays. This was done to avoid any bias arising from probe selection on the cDNA platform or from multiple probe-sets for individual genes on the Affymetrix platform.

For the integration of gene expression profiling data and biological processes regulated by RUNX1, genes were ranked in the test datasets by absolute moderated t-statistic. For the correlation with clinical AML samples, the test dataset was the previously published expression profiling data on 285 AML patients and 8 healthy individuals [23]. In this case, the Affymetrix probe-sets were ranked according to their correlation with the 11 RUNX1 probe-sets across the 293 RNA samples. Correlations were computed using Gene Recommender [61], which provides a very robust correlation measure suitable for this purpose. Probe-sets were also ranked by moderated t-statistic on their ability to distinguish the healthy patients from the 22 patients with t(8;21) or from the 18 patients with inv(16).

The MR-GSE p-values are computed by permuting genes rather than permuting arrays. This is necessary because the tests are designed for use with small numbers of arrays. The computation necessarily assumes that different genes have statistically independent expression values within experimental groups. When the gene set contains genes which are highly interdependent, and which vary substantially between biological replicates, the test may be anti-conservative. We checked the independence assumption for our data by computing average inter-gene correlations using REML. The inter-gene correlations were found to be generally very small at the expression level (data not shown), suggesting that the MS-GSE results are meaningful on our data.

### Bioinformatic identification of biological processes and cross-platform comparison

Enrichment of a gene ontology annotation in a dataset of differentially expressed genes compared to the genes present on the array was determined using the GOStat program [62]. For the MR-GSE test, relevant gene sets were taken from published reviews or independent microarray data (see Additional File 1: Table S4)

### BrdU proliferation assay

The Cell Proliferation ELISA, BrdU kit (Roche) was used to measure proliferation of cell lines derived from two independent families, including the family used for the microarray experiment (Pedigree 2) [9] and an additional family harboring a nonsense mutation Y260X present outside of the Runt domain (Pedigree 3, affected individuals III:7 and IV:4 and one unaffected individual III:8 [9]). Briefly, the cells were split into 96-well plates at an equal density. BrdU was added to the cells for 4 hours and the cells were then treated according to the manufacturer's protocol. The optical density (OD_450_) was measured on an ELISA plate reader. Technical triplicates and two independent experiments were performed. A two-way ANOVA (analysis of variance) test was performed.

### Tubulin polymerization assay

Soluble (cytosolic) and polymerized (cytoskeletal) fractions of tubulin were separated from the cell lines treated with or without 4 μg/ml of Taxol as described [63]. The same cell lines used for the proliferation assay were assessed. Results were expressed as a percentage of polymerized tubulin by dividing the densitometric value of polymerized tubulin (insoluble) by the total tubulin content (sum of densitometric value of soluble and polymerized tubulin). Three independent experiments were performed and a two-way ANOVA was done.

### Glycophorin A assay

Blood samples were collected in EDTA-tubes, with informed consent, from seven individuals heterozygous (MN phenotype) at the glycophorin A locus. These include: a FPD-AML patient harboring a frameshift mutation (N69fsX94) and her unaffected sister, a second FPD-AML patient harboring a nonsense mutation (Pedigree 3 (Y260X), individual IV:4) [9] and 4 independent unaffected individuals. The assay is described in detail in Additional File 1. A two-way ANOVA test was performed to compare the 5 controls to the 2 affected individuals.

### Luciferase reporter assay

Genomic regions overlapping the conserved binding sites (300–400 bps) were amplified from BACs and cloned into pGL3-Basic vector (Promega #E1751). Each construct was co-transfected into HeLa cells using lipofectamine 2000 (Invitrogen) along with pSCOT plasmids expressing RUNX1 and CBFβ or empty vector to keep the amount of plasmid constant. For normalization, 20 ng of pRL-TK vector (Renilla luciferase Promega #E2241) was also co-transfected. The luciferase activities were measured using the Dual-Luciferase Reporter Assay System (Promega #E1910). The increase or decrease in luciferase activity was determined as a function of the endogenous activity of each construct.

### cDNA panel production

The human cDNA panel was generated as described [26]. The relative amount of each cDNA was normalized according to housekeeping gene levels. More details are described in Additional File 1.

## Competing interests

The authors declare that they have no competing interests.

## Authors' contributions

JM designed the experiments and analysis, performed the majority of the experiments and wrote the manuscript. KMS performed the statistical analysis of the Affymetrix data and participated in the Gene Set Enrichment analysis. RE participated in the design of the experiments. KBP participated in the generation of adenovirus particles. TB participated in the design of the bioinformatics analyses. CC participated in the luciferase reporter assay. MER participated in the microarray analyses. FS performed the ANOVA tests and participated in the cross-platform comparison. PC participated in the luciferase reporter assay. ML performed the tubulin polymerization assay. XS performed the GPA assay. YI provided vital Runx1 knockout embryos. WHR provided vital patient samples. MSH provided vital patient samples. MO provided vital Runx1 knockout embryos. DRT participated in the design and analysis of the GPA assay. TPS participated in the design of the bioinformatics analyses. MK participated in the design and analysis of the tubulin polymerization assay. GKS generated the statistical analysis of the cDNA microarray data, the statistical comparison to AML samples and supervised the statistical components of the article. HSS designed the experiments and analysis and participated in the writing of the manuscript. All authors read and approved the final manuscript.

## Supplementary Material

Additional File 1 Additional Methods, Figures S1 to S4 and Tables S3 to S6. Additional figures and tables to support the statistical and bioinformatics analyses described in the manuscript. Click here for file

Additional File 2 Table S1. Gene expression profiling results. Summary of the gene expression profiling results, oPOSSUM and corresponding mouse Affymetrix data for each clone Accession number present on the human cDNA array. Click here for file

Additional File 3 Table S2. Gene expression profiling data for E8.5 and E12 *Runx1 *knockout embryos. Click here for file

Additional File 4 Figure S4. Supporting graphs for the Gene Set Enrichment analysis. Click here for file

Additional File 5 Figure S5. Expression pattern of RUNX1 and a subset of differentially expressed genes. Click here for file
