# Supplementary material for: Integrative analysis of RUNX1 downstream pathways and target genes
Source: BMC Genomics. 2008 Jul 31;9:363. doi: 10.1186/1471-2164-9-363 (PMC2529319; doi:10.1186/1471-2164-9-363)
Supplement: Additional File 1 — Additional Methods, Figures S1 to S4 and Tables S3 to S6. Additional figures and tables to support the statistical and bioinformatics analyses described in the manuscript. [file 1471-2164-9-363-S1.doc]

# Additional Information for Michaud et al. 2008

1. Additional Material and Methods
2. Additional Table 1 legend: Summary of the gene expression profiling results, oPOSSUM and corresponding mouse Affymetrix data for each clone Accession number present on the human cDNA array.
3. Additional Table 2 legend: Gene expression profiling data for E8.5 and E12 *Runx1* knockout embryos.
4. Additional Table 3: Genes differentially expressed in the FPD-AML samples and also differentially expressed in t(8;21) and Inv(16) samples
5. Additional Table 4: Set of genes used for the Gene Set Enrichment analysis
6. Additional Table 5: List of genes differentially expressed in FPD and CBF, which are associated to cellular proliferation
7. Additional Table 6: List of genes differentially expressed following overexpression of the CBF complex and associated to the cytoskeleton
8. Additional Figure 1: Level of RUNX1 expression in FPD-AML cells
9. Additional Figure 2: protein overexpression of RUNX1 and CBFβ
10. Additional Figure 3: Correspondence analysis between gene expression profiles.
11. Additional Figure 4: Supporting graphs for the Gene Set Enrichment analysis
12. Additional Figure 5: Expression pattern of RUNX1 and a subset of differentially expressed genes
13. References for Additional Information

###### I. Additional Material and Methods

**Adenovirus production**

Recombinant adenoviruses were generated as described[1], except that VmRL-CMV1 and pSCOT were used as the adenovirus backbone and transfer vector respectively. VmRL-CMV1 is identical to VmAdcDNA3 except that it contains a beta-globin polyadenylation signal. The pSCOT vector contains two Tet operators flanking the TATA box allowing suppression of expression in the presence of Tet Repressors. The *RUNX1* p49 isoform[2] and *CBFβ* coding sequences were amplified from fetal lung cDNA and cloned into the pSCOT vector using EcoRV. The *EGFP* coding sequence was subcloned into pSCOT using NotI and SalI. Recombinant adenovirus was transfected into E1-complementing packaging cells HER911[3] and high titer of infectious particles were produced as described.[1] The titer of each adenovirus was measured by optical densitometry.

#### Identification of the genes and combination of human and mouse platforms

The genes corresponding to each cDNA clone were determined using Unigene (Build 181, 9/3/05). The EntrezGene IDs were obtained for each Unigene and Homologene (Build 39.2) was used to identify the mouse orthologous genes. Similarly Unigene cluster ID was assigned to each mouse Affymetrix probe (Affymetrix annotations 14 March 2005). Refseq IDs (UCSC May 2004) corresponding to each Unigene cluster were identified. More than one Unigene or Refseq number can correspond to the same cDNA clone.

**Glycophorin A assay**

Spheroplasts were prepared and frozen until required. Three to five technical replicates were generated for each sample. After thawing, spheroplasts were labeled with anti-GPA-M and anti-GPA-N antibodies and subjected to flow cytometry analysis [4], recording 2.5x10^6^ gated events for each sample. Spheroplasts with phenotypes N0 and NN were gated to have intensity ≤ 10% of the geometric mean M-labelled intensity for the wild-type MN population. N0 cells were gated to be centred on the MN geometric mean N intensity with gate width encompassing 95% of all cells.  NN cells were centred around twice the N0 mean with gates at the equivalent geometric fluorescence intensity width.

**oPOSSUM analysis (**[www.cisreg.ca/cgi-bin/oPOSSUM/opossum](http://www.cisreg.ca/cgi-bin/oPOSSUM/opossum)**)**

The oPOSSUM analysis was performed using 1) the top 30% of conserved regions with a minimum of 60% of conservation between human and mouse sequences, 2) a matrix match score of 80% and 3) non-coding sequences 5000bp upstream or downstream of the transcription start.

**cDNA panel production**

The human cDNA panel was generated as described.[5] Briefly cDNA was synthesized from 1μg of polyA RNA and normalized according to GAPDH level. A normalized dilution of 1:500 of the initial cDNA sample was used in PCR amplification. The human hematopoietic cell line cDNA panel was generated following a similar protocol except that 5μg of total RNA was reverse transcribed and the relative amount of each cDNA was normalized according to GAPDH and HPRT housekeeping gene levels determined using the QuantiTect SYBR Green PCR kit and the Lightcycler PCR machine. A normalized dilution of 1:250 of the initial cDNA sample was used in PCR amplification.

II.

**Additional** **Table 1: Human datasets.** A summary of all the human data obtained in this study and the corresponding mouse Affymetrix probes are indicated in this table. The Unigene cluster ID, gene symbol and gene name are indicated for each clone accession number present on the array according to Unigene Build 181. Each human dataset (FPD and CBF) is represented by three columns: the first column is a classification column representing whether the gene is up- (1), down- (-1) or not differentially expressed in the mutant following statistical analysis as described above. The second and third columns represent the M-value (log_2_ of the fold change) and the moderated t-statistics, respectively. The following column contains the corresponding Refseq numbers. The presence or absence of a RUNX1 binding site in the regulatory region of the gene is then indicated by “1” or “0” respectively (oPOSSUM). ND means that the regulatory region of the gene could not be identified in the oPOSSUM database. The next column gives the corresponding Human Gene IDs (March 2005). The corresponding mouse Affymetrix probes were identified using Gene IDs and Homologene (Build 39.2). The Unigene cluster IDs corresponding to each Affymetrix probe were obtained from the latest Affymetrix annotation file (March 2005). M and t are also showed for the mouse probesets.

III.

**Additional** **Table 2: Mouse datasets.** The M (log_2_ of the fold change) and moderated t-statistics for each Affymetrix probe is indicated as well as the identity of the probes according to the latest Affymetrix annotation file (March 2005).

IV.

Additional Table 3: Top 14 differentially expressed genes in the FPD-AML cells that are also differentially expressed in t(8;21) and Inv(16) samples.

| **FPD_t(8;21)** | | | | |
| --- | --- | --- | --- | --- |
| Gene Symbol | Gene Title | FPD M | Probe Set ID | rank in t(8;21) samples |
| TAF9 | TAF9 RNA polymerase II, TATA box binding protein (TBP)-associated factor, 32kDa | 0.22 | 203893_at | 84 |
| SILV | silver homolog (mouse) | -0.27 | 209848_s_at | 133 |
| SHOX2 | short stature homeobox 2 | 0.69 | 210134_x_at | 140 |
| CD83 | CD83 molecule | 0.39 | 204440_at | 166 |
| PPIB | peptidylprolyl isomerase B (cyclophilin B) | -0.29 | 200967_at | 245 |
| ARPC5 | actin related protein 2/3 complex, subunit 5, 16kDa | 0.24 | 211963_s_at | 276 |
| HLA-DQB1 | major histocompatibility complex, class II, DQ beta 1 | 0.35 | 211654_x_at | 299 |
| MT1G | metallothionein 1G | -0.73 | 210472_at | 397 |
| LIPG | lipase, endothelial | -0.26 | 219181_at | 574 |
| TCF12 | transcription factor 12 (HTF4, helix-loop-helix transcription factors 4) | 0.17 | 215611_at | 630 |
| CALR | calreticulin | -0.38 | 214315_x_at | 792 |
| PHYH | phytanoyl-CoA 2-hydroxylase | -0.24 | 203335_at | 797 |
| IMPA2 | inositol(myo)-1(or 4)-monophosphatase 2 | -0.47 | 203126_at | 829 |
| KDELR2 | KDEL (Lys-Asp-Glu-Leu) endoplasmic reticulum protein retention receptor 2 | -0.23 | 200698_at | 903 |
| **FPD_Inv(16)** | | | | |
| Gene Symbol | Gene Title | FPD M | Probe Set ID | rank in Inv(16) samples |
| NFKBIA | nuclear factor of kappa light polypeptide gene enhancer in B-cells inhibitor, alpha | 0.24 | 201502_s_at | 536 |
| CD2BP2 | CD2 (cytoplasmic tail) binding protein 2 | -0.26 | 202257_s_at | 919 |
| SNAP25 | synaptosomal-associated protein, 25kDa | -0.37 | 202508_s_at | 965 |
| RGS1 | regulator of G-protein signalling 1 | 0.2 | 202988_s_at | 1110 |
| GOLPH4 | golgi phosphoprotein 4 | 0.35 | 204322_at | 911 |
| ACAT1 | acetyl-Coenzyme A acetyltransferase 1 (acetoacetyl Coenzyme A thiolase) | 0.3 | 205412_at | 389 |
| CD38 | CD38 molecule | -0.67 | 205692_s_at | 801 |
| UBD | ubiquitin D | 0.67 | 205890_s_at | 944 |
| CLTB | clathrin, light polypeptide (Lcb) | 0.23 | 206284_x_at | 469 |
| MT1X | metallothionein 1X | -0.69 | 208581_x_at | 447 |
| IGF1 | insulin-like growth factor 1 (somatomedin C) | -0.41 | 209540_at | 746 |
| ELF1 | E74-like factor 1 (ets domain transcription factor) | -0.38 | 212418_at | 1060 |
| PRKD3 | protein kinase D3 | -0.4 | 218236_s_at | 207 |
| UNC13B | Unc-13 homolog B (C. elegans) | -0.36 | 221130_s_at | 148 |

V.

**Additional** **Table 4. Set of genes used for the Gene Set Enrichment analysis.** Reference and description of each study, the format of the available data, the platform used in the study and the number of corresponding Unigene cluster ID (Build 181) are indicated. DEG: differentially expressed genes. M: Log_2_ of the fold change. A: Log_2_ of the overall intensity of the probe.

| **Gene set name** | **Reference** | **Study** | **Data format** | **Arrays used in the study** | **Number of Unigene clusters** |
| --- | --- | --- | --- | --- | --- |
| **Megakaryopoiesis** |  |  |  |  |  |
| Mekagaryocyte differentiation | [6] | Identification of genes involved in the differentiation of megakaryocytes. DEGs between stem cells and differentiated megakaryocytes | List of DEGs | U95Av2 | 103 |
| Platelets | [7] | Transcription profiling of human blood platelet | Top 50 DEGs | U95-Av2 | 36 |
| Normal megakaryocytes | [8] | Genes highly expressed in megakaryocytes | Top 200 genes with high expression | U133 | 113 |
| ET megakaryocytes | [8] | Genes highly expressed in essential thrombocytopenia megakaryocytes | Top 200 genes with high expression | U133 | 117 |
| ET vs normal | [8] (GDS552) | DEGs between normal megakaryocytes and megakaryocytes derived from patients with essential thrombocytopenia (ET) | Genes with a |M|>2 and A>4 | U133 | 128 |
| **Cell cycle** |  |  |  |  |  |
| Cytokinesis proteome | [9] | Identification of proteins present in the midbody during cytokinesis | List of proteins | Mass spectrometry | 155 |
| Spindle checkpoint | [10] | Review |  |  | 12 |
| **Genomic instability** |  |  |  |  |  |
| DNA repair | [11] | Review |  |  | 127 |
| Lymphoblast irradiation; high dose | [12] (GDS479) | Effect of ionising radiation on lymphoblasts | List of DEGs | U95A | 625 |
| Lymphoblast irradiation; low dose | [12] (GDS479) | Effect of ionising radiation on lymphoblasts | List of DEGs | U95A | 243 |
| **Cancer associated genes** |  |  |  |  |  |
| Genes DE in cancer | [13] | Meta-analysis of cancer microarray data to identify genes consistently DE in tumours | List of genes | Many | 67 |

VI.

**Additional** **Table 5**: List of genes differentially expressed in FPD and CBF, which are associated to cellular proliferation using Gene Ontology annotation GO:0008283. The M-values are shown for both FPD and CBF datasets. Genes in bold are those identified as differentially expressed in both datasets.

| **Accession** | **Symbol** | **RefSeq** | **FPD M** | **CBF M** |
| --- | --- | --- | --- | --- |
| W69954 | AIF1 | NM_001623 | 0.68 | 0.11 |
| AA857163 | AREG | NM_001657 | -0.36 | -0.01 |
| T69273 | ATPIF1 | NM_178191 | 0.25 | 0.09 |
| AI341427 | BCAT1 | NM_005504 | 0.37 | -0.14 |
| AA132086 | C6ORF108 | NM_199184 | 0.31 | -0.01 |
| AI375736 | CD28 | NM_006139 | 0.86 | 0.03 |
| AA973397 | CD86 | NM_006889 | -0.67 | -0.02 |
| N62245 | CDC7 | NM_003503 | 0.27 | -0.07 |
| AA873604 | CRIP1 | NM_001311 | -0.3 | 0.08 |
| AA878880 | CXCL10 | NM_001565 | 1.34 | -0.03 |
| AA450009 | EDNRA | NM_001957 | 0.5 | 0.11 |
| R54846 | FGFR1 | NM_015850 | 1.17 | 0.12 |
| AA448277 | FOXO1A | NM_002015 | -0.43 | 0.07 |
| N67876 | IGF1 | NM_000618 | -0.41 | 0.01 |
| AA977194 | IL12RB2 | NM_001559 | 0.3 | -0.07 |
| AA479795 | ISG20 | NM_002201 | -0.54 | -0.01 |
| AA045731 | KLF10 | NM_005655 | -0.35 | 0.18 |
| AA826328 | MALT1 | NM_006785 | -1.38 | 0.08 |
| AA705886 | MXI1 | NM_130439 | -0.33 | -0.01 |
| W56300 | NFKBIA | NM_020529 | 0.24 | -0.01 |
| AA935262 | NODAL | NM_018055 | -0.82 | -0.01 |
| AA701502 | PDGFA | NM_002607 | 0.36 | -0.06 |
| AA863383 | PIM2 | NM_006875 | -0.62 | 0.01 |
| N89985 | PURB | NM_033224 | -0.3 | -0.02 |
| AA458996 | SLAMF1 | NM_003037 | -0.03 | 0.4 |
| W72201 | SMAD3 | NM_005902 | -0.31 | -0.07 |
| AA481026 | SMARCA2 | NM_003070 | 0.57 | 0.01 |
| AA007444 | TLX1 | NM_005521 | -0.7 | 0.07 |
| AA166695 | TNFSF13B | NM_006573 | 0.27 | 0 |
| AA486088 | TOB2 | NM_016272 | -0.26 | 0.06 |
| AI950056 | TPX2 | NM_012112 | 0.23 | 0.01 |
| H63077 | **ANXA1** | NM_000700 | 0.93 | -0.4 |
| AA670438 | **UCHL1** | NM_004181 | -1.12 | -0.25 |
| AA490477 | **MYH10** | NM_005964 | 0.45 | 0.12 |
| AA858175 | **RUNX2** | NM_004348 | -0.46 | 0.53 |
| AA968896 | MDK | NM_002391 | -0.1 | 0.3 |
| W15277 | TBC1D8 | NM_007063 | 0.1 | 0.18 |
| AA287300 | CDC2L1 | NM_033489 | -0.08 | 0.29 |
| N45138 | TGFB2 | NM_003238 | -0.05 | 0.37 |
| AA865712 | FGFR1OP | NM_194429 | -0.1 | 0.26 |
| AA167269 | NAP1L1 | NM_139207 | 0.24 | 0.13 |
| H07899 | VEGFC | NM_005429 | 0.03 | 0.12 |
| T59334 | CSRP2 | NM_001321 | -0.06 | 0.46 |
| AI828088 | CDKN1C | NM_000076 | -0.04 | 0.08 |
| AA447661 | SESN1 | NM_014454 | -0.27 | 0.26 |
| AA102526 | IL8 | NM_000584 | 0.24 | 0.17 |
| AA996024 | HDAC4 | NM_006037 | -0.14 | 0.1 |
| N72115 | CDKN2C | NM_001262 | 0.25 | 0.2 |
| N20203 | BMPR2 | NM_001204 | -0.24 | 0.38 |
| AA130714 | PGF | NM_002632 | 0.2 | 0.27 |
| N20338 | HGS | NM_004712 | -0.09 | 0.14 |
| R43576 | BLZF1 | NM_003666 | -0.14 | 0.33 |
| AA040427 | CLK1 | NM_004071 | -0.19 | 0.16 |

VII.

#### Additional Table 6: Cytoskeleton–related differentially expressed genes in CBF dataset. 57 differentially expressed genes following overexpression of CBF complex are associated to the cytoskeleton using Gene Ontology annotation GO:0005875 (cellular component). The corresponding gene symbols and gene names are indicated (Unigene Build 181). The direction of the difference in expression observed following overexpression of CBF is also indicated.

| Accession | Gene symbol | Gene name | Difference in expression in CBF |
| --- | --- | --- | --- |
| AA001749 | MAPRE1 | Microtubule-associated protein, RP/EB family, member 1 | Down |
| AA151125 | TMOD3 | Tropomodulin 3 (ubiquitous) | Down |
| AA180742 | TUBA1 | Tubulin, alpha 1 (testis specific) | Down |
| AA406601 | ABLIM1 | Actin binding LIM protein 1 | Down |
| AA424824 | DSTN | Destrin (actin depolymerizing factor) | Down |
| AA426374 | TUBA2 | Tubulin, alpha 2 | Down |
| AA430574 | PXN | Paxillin | Down |
| AA431967 | LATS2 | LATS, large tumor suppressor, homolog 2 (Drosophila) | Down |
| AA432066 | SGCE | Sarcoglycan, epsilon | Down |
| AA436460 | KIFC3 | Kinesin family member C3 | Down |
| AA446462 | BUB1 | BUB1 budding uninhibited by benzimidazoles 1 homolog (yeast) | Down |
| AA449336 | PRC1 | Protein regulator of cytokinesis 1 | Down |
| AA459400 | ARHGDIA | Rho GDP dissociation inhibitor (GDI) alpha | Down |
| AA460685 | BIRC5 | Baculoviral IAP repeat-containing 5 (survivin) | Down |
| AA485959 | KRT7 | Keratin 7 | Down |
| AA488676 | BASP1 | Brain abundant, membrane attached signal protein 1 | Down |
| AA496691 | DAG1 | Dystroglycan 1 (dystrophin-associated glycoprotein 1) | Down |
| AA496785 | ABL1 | V-abl Abelson murine leukemia viral oncogene homolog 1 | Down |
| AA504128 | RAE1 | RAE1 RNA export 1 homolog (S. pombe) | Down |
| AA599145 | ZW10 | ZW10 homolog, centromere/kinetochore protein (Drosophila) | Down |
| AA621315 | CTNNAL1 | Catenin (cadherin-associated protein), alpha-like 1 | Down |
| AA630298 | PTK2 | PTK2 protein tyrosine kinase 2 | Down |
| AA634006 | ACTA2 | Actin, alpha 2, smooth muscle, aorta | Down |
| AA634289 | ACTL7B | Actin-like 7B | Down |
| AA676955 | RHOA | Ras homolog gene family, member A | Down |
| AA865469 | TUBA3 | Tubulin, alpha 3 | Down |
| AA873060 | STMN1 | Stathmin 1/oncoprotein 18 | Down |
| AA888148 | TUBB2 | Tubulin, beta, 2 | Down |
| H63077 | ANXA1 | Annexin A1 | Down |
| H98666 | PCOLN3 | Procollagen (type III) N-endopeptidase | Down |
| N74524 | TUBB4 | Tubulin, beta 4 | Down |
| R16712 | ANLN | Anillin, actin binding protein (scraps homolog, Drosophila) | Down |
| R76544 | HAX1 | HS1 binding protein | Down |
| R77252 | MAP7 | Microtubule-associated protein 7 | Down |
| T60048 | ACTG2 | Actin, gamma 2, smooth muscle, enteric | Down |
| T61428 | NEDD9 | Neural precursor cell expressed, developmentally down-regulated 9 | Down |
| T77733 | TUBG1 | Tubulin, gamma 1 | Down |
| W72207 | CSTA | Cystatin A (stefin A) | Down |
| W95389 | SHD1 | Sac3 homology domain 1 (S. cerevisiae) | Down |
| AA001897 | SPTA1 | Spectrin, alpha, erythrocytic 1 (elliptocytosis 2) | Up |
| AA025276 | CTNND1 | Catenin (cadherin-associated protein), delta 1 | Up |
| AA057796 | MYO1B | Myosin IB | Up |
| AA284634 | JAK1 | Janus kinase 1 (a protein tyrosine kinase) | Up |
| AA461325 | ADD3 | Adducin 3 (gamma) | Up |
| AA482231 | MARCKS | Myristoylated alanine-rich protein kinase C substrate | Up |
| AA490477 | MYH10 | Myosin, heavy polypeptide 10, non-muscle | Up |
| AA495981 | ARHGAP6 | Rho GTPase activating protein 6 | Up |
| AA679180 | PTPN13 | Protein tyrosine phosphatase, non-receptor type 13 (APO-1/CD95 (Fas)-associated phosphatase) | Up |
| AA774983 | TPM4 | Tropomyosin 4 | Up |
| AI261600 | WASL | Wiskott-Aldrich syndrome-like | Up |
| AW005820 | PTPN4 | Protein tyrosine phosphatase, non-receptor type 4 (megakaryocyte) | Up |
| AW075457 | CCT3 | Chaperonin containing TCP1, subunit 3 (gamma) | Up |
| R56096 | WASF1 | WAS protein family, member 1 | Up |
| T55835 | HDAC6 | Histone deacetylase 6 | Up |
| T57805 | ROCK1 | Rho-associated, coiled-coil containing protein kinase 1 | Up |
| W42849 | APP | Amyloid beta (A4) precursor protein (protease nexin-II, Alzheimer disease) | Up |
| W86182 | PNN | Pinin, desmosome associated protein | Up |
|  |  |  |  |

VIII.


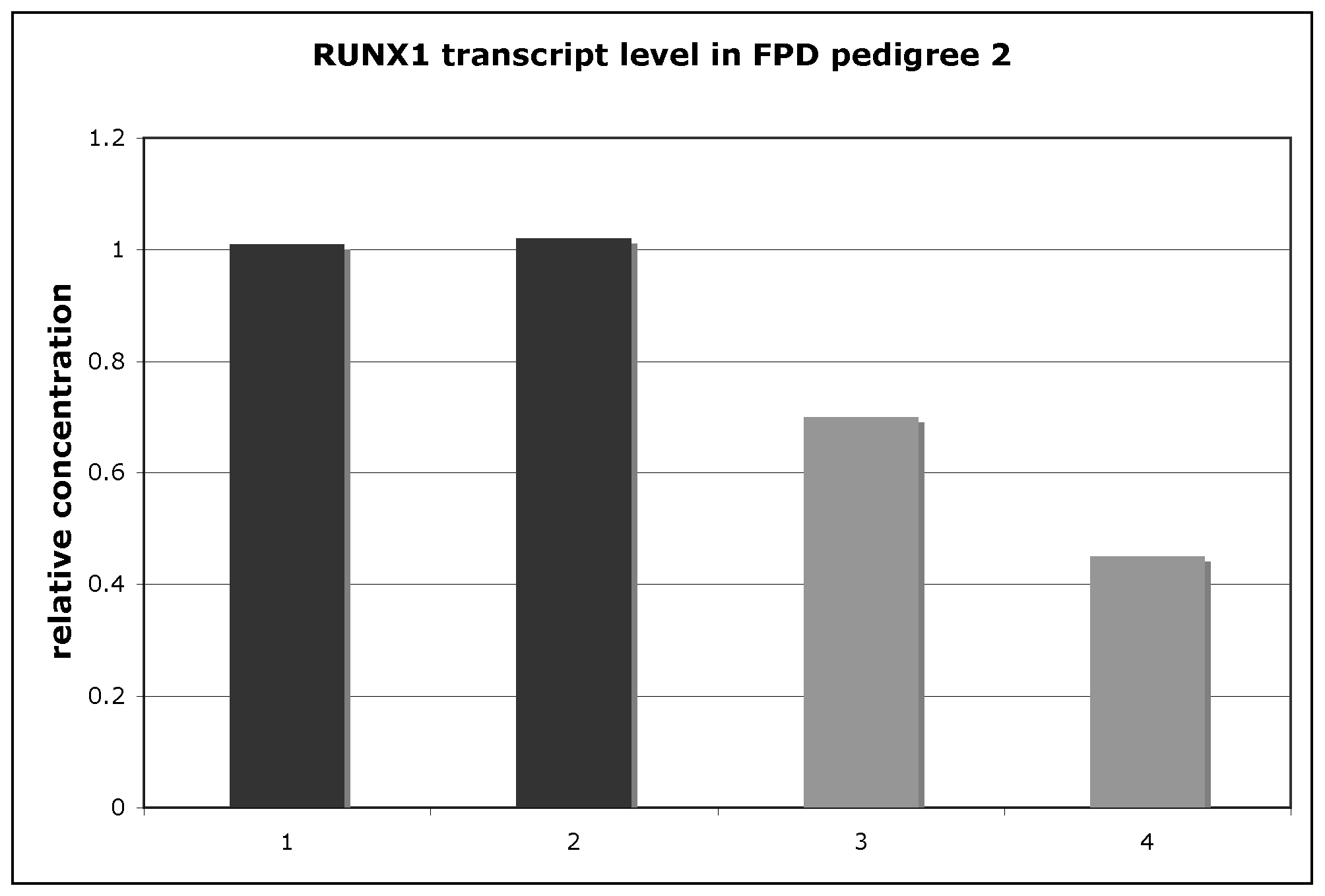


**Additional** **Figure 1:** The level of the RUNX1 transcript was measured by quantitative RT-PCR using the lightcycler PCR machine (Roche). The concentration of the RUNX1 transcript is relative to the level of the housekeeping gene PSMB2. The two affected individuals (3, 4) show a decrease of expression compared to the unaffected individuals (1, 2). 1: IV:1, 2: V:3, 3: V:1, 4: V:2, as described in [14].

IX.


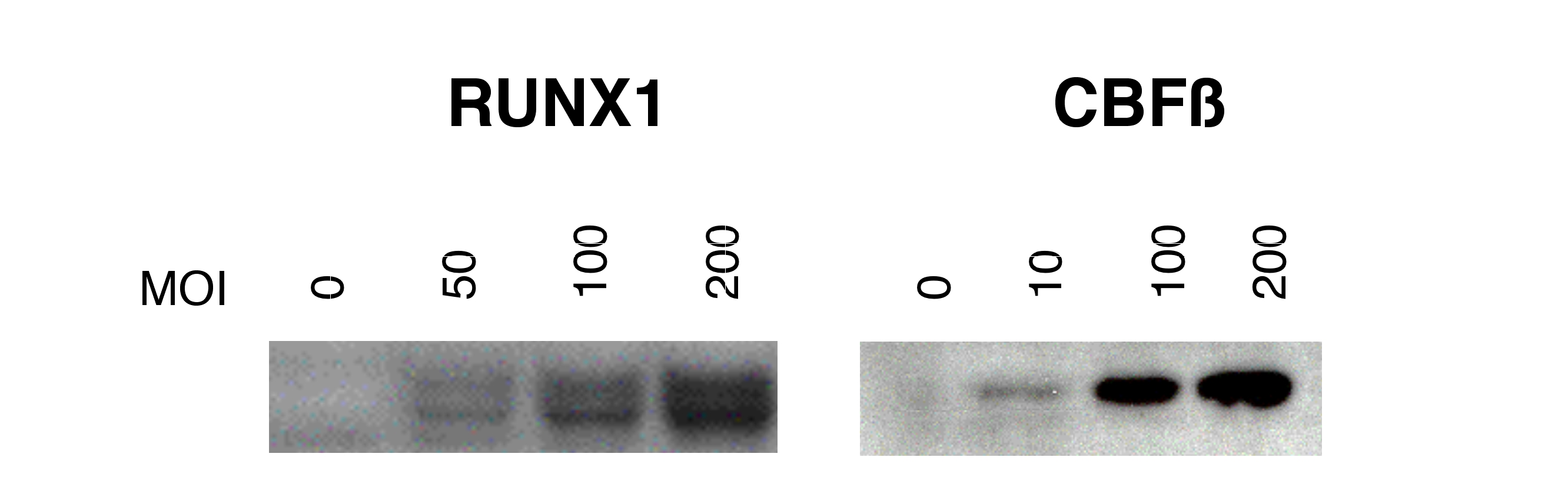


**Additional** **Figure 2: protein overexpression of RUNX1 and CBFβ**.

Levels of exogenous proteins were determined by western blot in Hela cells following infection with a range of multiplicity of infection (MOI) of adenovirus. The antibodies used in this assay do not recognize the endogenous proteins.

X.


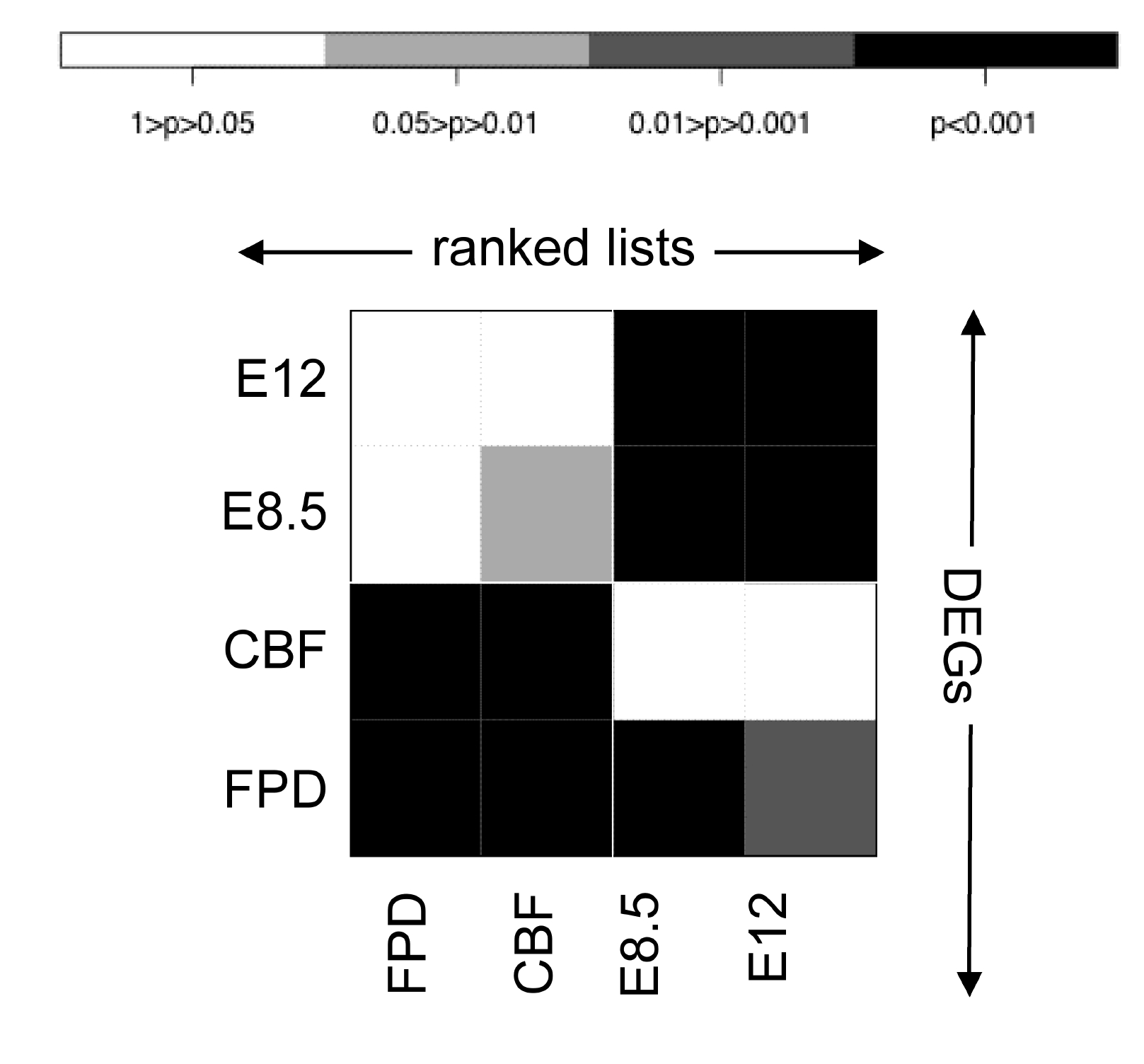


**Additional** **Figure 3: Correspondence between datasets of differentially expressed genes (DEGs).** A mean-rank gene set enrichment test was performed to determine the rank of the DEGs in the ranked data (absolute t-statistics) of the other approaches. Significant p-values mean that a high correlation was observed between these approaches. The p-values are corrected for multiple testing. FPD: FPD-AML dataset, CBF: overexpression dataset, E8.5 and E12: embryonic stages for the mouse datasets, DEG: differentially expressed genes.

XI.

**Additional** **Figure 4. Gene Set Enrichment analysis.** Histograms of normalized rank for each gene set. Sets with adjusted p-value lower than 0.05 are plotted in red. Normalized ranks were calculated by dividing the rank of each gene by the total number of genes. The value plotted on the y-axis is the percentage of genes, which lies in each bin, and is calculated by dividing the counts in the bin by the total number of genes in the gene set.

XII.

**Additional** **Figure 5. Expression patterns of RUNX1 and a subset of differentially expressed genes.** The left panel represents a cDNA panel containing 20 human tissues and the right panel contains hematopoietic cell lines. RUNX1 expression is shown at the top. A number of RUNX1 isoforms were identified. The genes present in the first half of the figure are strongly expressed in the hematopoietic cells whereas the second half shows a low expression in these cells. Actin expression is shown at the bottom to illustrate the presence of an equal amount of template in each well.

**XIII. References for Additional Information**

1. Chaussade C, Pirola L, Bonnafous S, Blondeau F, Brenz-Verca S, Tronchere H, Portis F, Rusconi S, Payrastre B, Laporte J *et al*: **Expression of myotubularin by an adenoviral vector demonstrates its function as a phosphatidylinositol 3-phosphate [PtdIns(3)P] phosphatase in muscle cell lines: involvement of PtdIns(3)P in insulin-stimulated glucose transport**. *Mol Endocrinol* 2003, **17**(12):2448-2460.

2. Levanon D, Glusman G, Bangsow T, Ben-Asher E, Male DA, Avidan N, Bangsow C, Hattori M, Taylor TD, Taudien S *et al*: **Architecture and anatomy of the genomic locus encoding the human leukemia-associated transcription factor RUNX1/AML1**. *Gene* 2001, **262**(1-2):23-33.

3. Fallaux FJ, Kranenburg O, Cramer SJ, Houweling A, Van Ormondt H, Hoeben RC, Van Der Eb AJ: **Characterization of 911: a new helper cell line for the titration and propagation of early region 1-deleted adenoviral vectors**. *Hum Gene Ther* 1996, **7**(2):215-222.

4. Jensen RH, Bigbee WL: **Direct immunofluorescence labeling provides an improved method for the glycophorin A somatic cell mutation assay**. *Cytometry* 1996, **23**(4):337-343.

5. Michaud J, Kudoh J, Berry A, Bonne-Tamir B, Lalioti MD, Rossier C, Shibuya K, Kawasaki K, Asakawa S, Minoshima S *et al*: **Isolation and characterization of a human chromosome 21q22.3 gene (WDR4) and its mouse homologue that code for a WD-repeat protein**. *Genomics* 2000, **68**(1):71-79.

6. Shim MH, Hoover A, Blake N, Drachman JG, Reems JA: **Gene expression profile of primary human CD34+CD38lo cells differentiating along the megakaryocyte lineage**. *Exp Hematol* 2004, **32**(7):638-648.

7. Gnatenko DV, Dunn JJ, McCorkle SR, Weissmann D, Perrotta PL, Bahou WF: **Transcript profiling of human platelets using microarray and serial analysis of gene expression**. *Blood* 2003, **101**(6):2285-2293.

8. Tenedini E, Fagioli ME, Vianelli N, Tazzari PL, Ricci F, Tagliafico E, Ricci P, Gugliotta L, Martinelli G, Tura S *et al*: **Gene expression profiling of normal and malignant CD34-derived megakaryocytic cells**. *Blood* 2004, **104**(10):3126-3135.

9. Skop AR, Liu H, Yates J, 3rd, Meyer BJ, Heald R: **Dissection of the mammalian midbody proteome reveals conserved cytokinesis mechanisms**. *Science* 2004, **305**(5680):61-66.

10. Lew DJ, Burke DJ: **The spindle assembly and spindle position checkpoints**. *Annu Rev Genet* 2003, **37**:251-282.

11. Wood RD, Mitchell M, Sgouros J, Lindahl T: **Human DNA repair genes**. *Science* 2001, **291**(5507):1284-1289.

12. Jen KY, Cheung VG: **Transcriptional response of lymphoblastoid cells to ionizing radiation**. *Genome Res* 2003, **13**(9):2092-2100.

13. Rhodes DR, Yu J, Shanker K, Deshpande N, Varambally R, Ghosh D, Barrette T, Pandey A, Chinnaiyan AM: **Large-scale meta-analysis of cancer microarray data identifies common transcriptional profiles of neoplastic transformation and progression**. *Proc Natl Acad Sci U S A* 2004, **101**(25):9309-9314.

14. Michaud J, Wu F, Osato M, Cottles GM, Yanagida M, Asou N, Shigesada K, Ito Y, Benson KF, Raskind WH *et al*: **In vitro analyses of known and novel RUNX1/AML1 mutations in dominant familial platelet disorder with predisposition to acute myelogenous leukemia: implications for mechanisms of pathogenesis**. *Blood* 2002, **99**(4):1364-1372.
