# Supplementary figures and images for: Integrative analysis of RUNX1 downstream pathways and target genes
Source: BMC Genomics. 2008 Jul 31;9:363. doi: 10.1186/1471-2164-9-363 (PMC2529319; doi:10.1186/1471-2164-9-363)

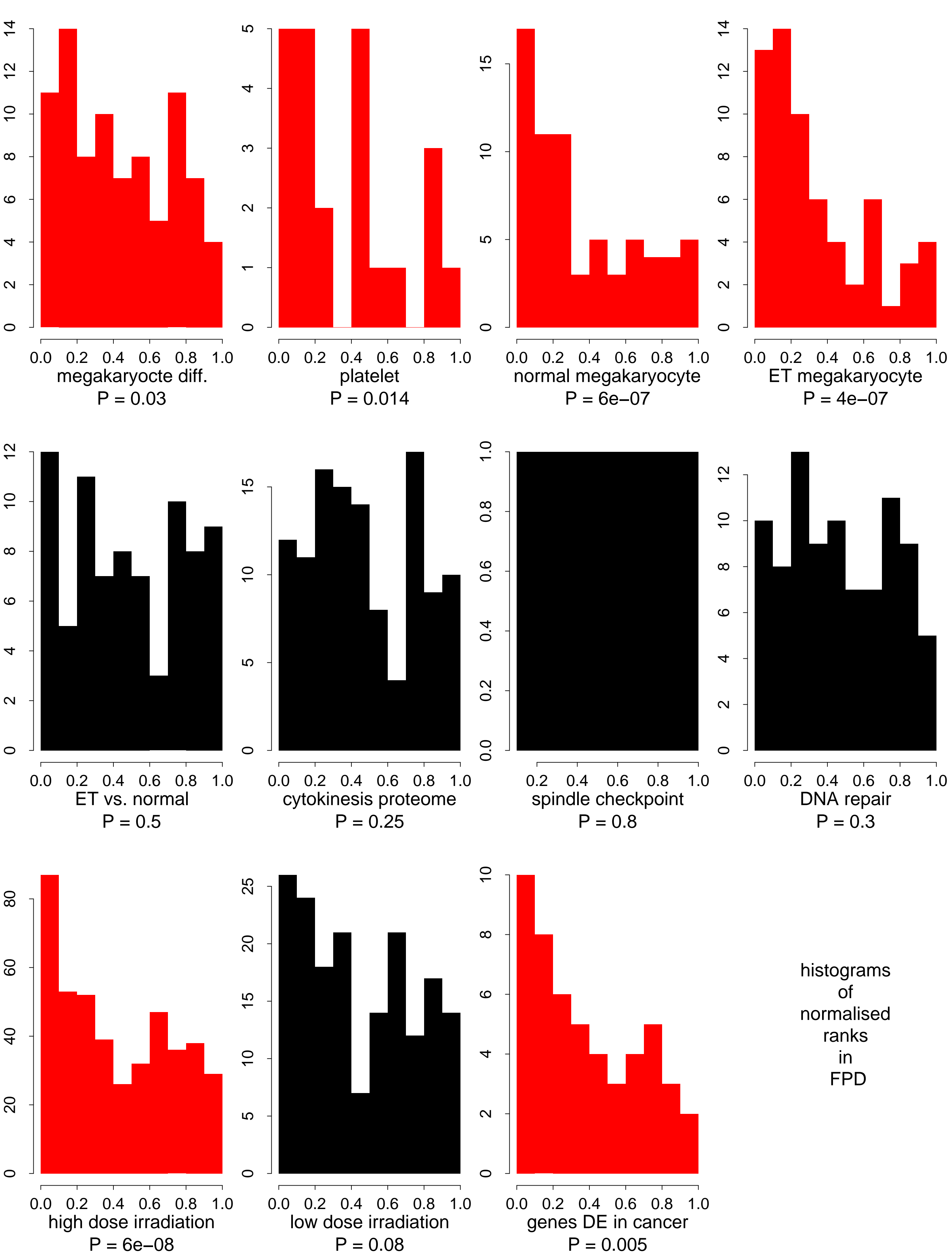

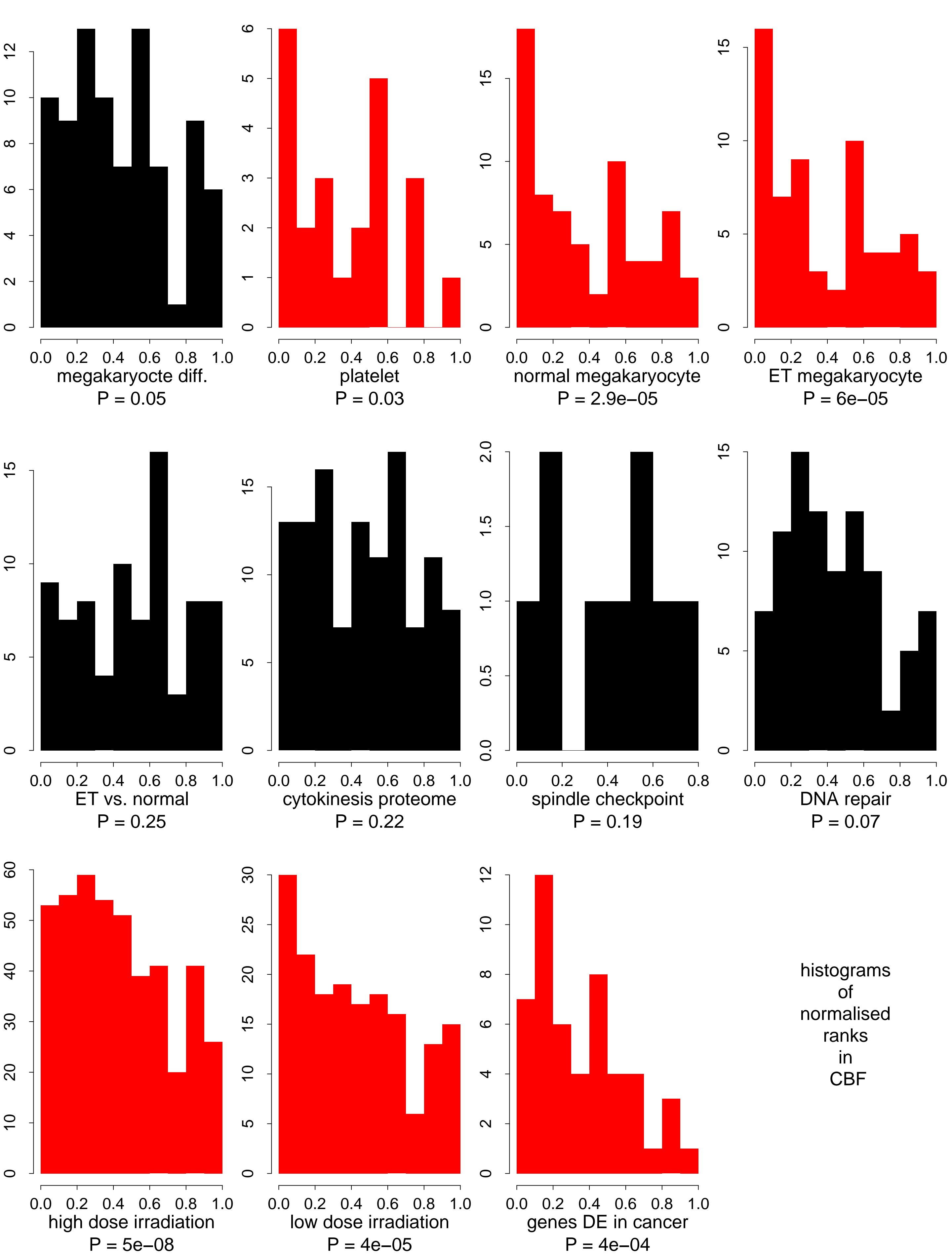

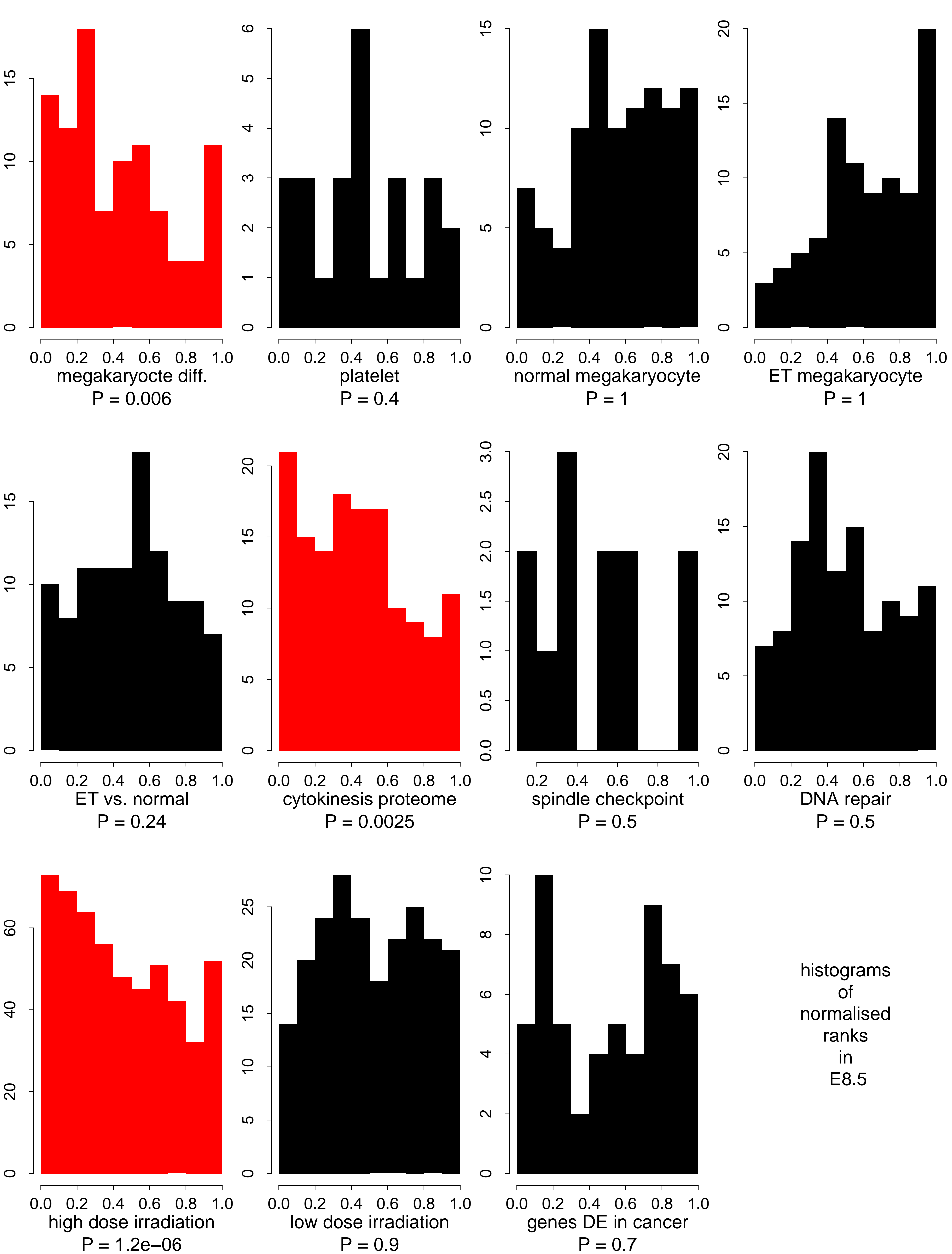

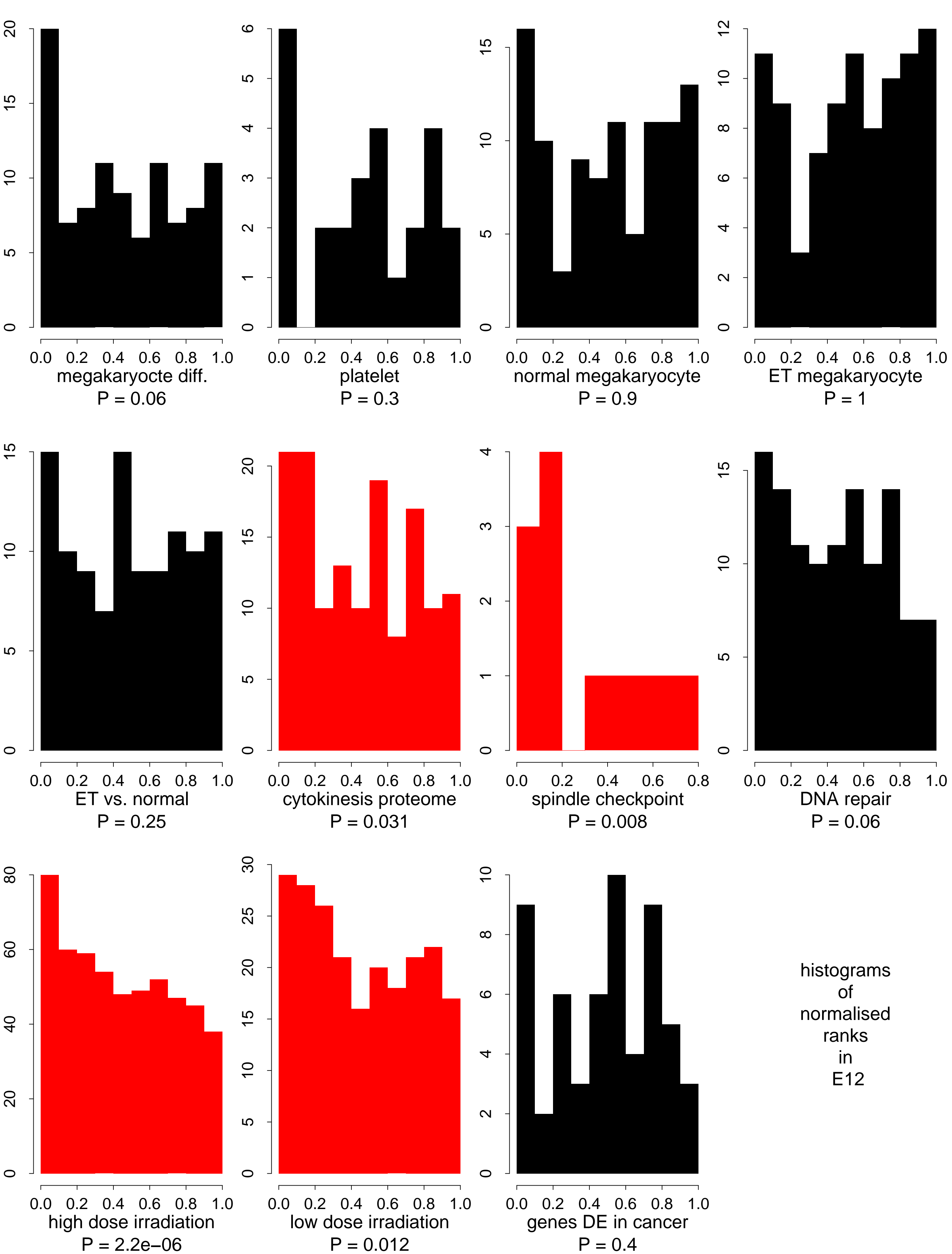

Supplement: Additional File 4 — Figure S4. Supporting graphs for the Gene Set Enrichment analysis. [file 1471-2164-9-363-S4.pdf]

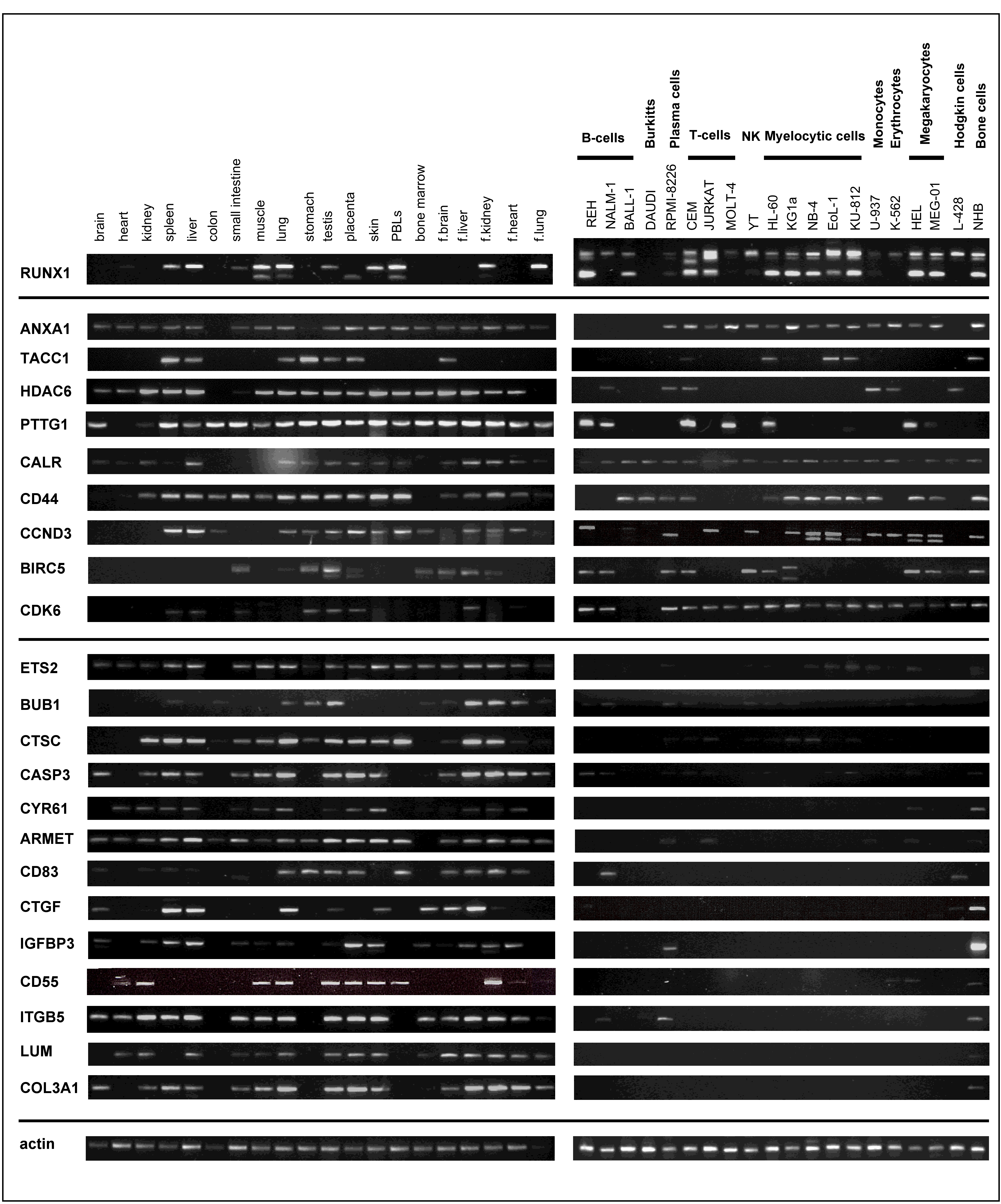

Supplement: Additional File 5 — Figure S5. Expression pattern of RUNX1 and a subset of differentially expressed genes. [file 1471-2164-9-363-S5.png]
